# Phylogenomic early warning signals for SARS-CoV-2 epidemic waves

**DOI:** 10.1016/j.ebiom.2023.104939

**Published:** 2024-01-08

**Authors:** Kieran O. Drake, Olivia Boyd, Vinicius B. Franceschi, Rachel M. Colquhoun, Nicholas A.F. Ellaby, Erik M. Volz

**Affiliations:** a MRC Centre for Global Infectious Disease Analysis, Department of Infectious Disease Epidemiology, School of Public Health, Imperial College London, London, United Kingdom; b Institute of Evolutionary Biology, Ashworth Laboratories, University of Edinburgh, Edinburgh, United Kingdom; c UK Health Security Agency, London, United Kingdom

**Keywords:** SARS-CoV-2, COVID-19, Early warning signal, Leading indicator, Surveillance, Phylogenetics, Epidemic, Infectious disease

## Abstract

**Background:**

Epidemic waves of coronavirus disease 2019 (COVID-19) infections have often been associated with the emergence of novel severe acute respiratory syndrome coronavirus 2 (SARS-CoV-2) variants. Rapid detection of growing genomic variants can therefore serve as a predictor of future waves, enabling timely implementation of countermeasures such as non-pharmaceutical interventions (social distancing), additional vaccination (booster campaigns), or healthcare capacity adjustments. The large amount of SARS-CoV-2 genomic sequence data produced during the pandemic has provided a unique opportunity to explore the utility of these data for generating early warning signals (EWS).

**Methods:**

We developed an analytical pipeline (Transmission Fitness Polymorphism Scanner – designated in an R package *mrc-ide/tfpscanner*) for systematically exploring all clades within a SARS-CoV-2 virus phylogeny to detect variants showing unusually high growth rates. We investigated the use of these cluster growth rates as the basis for a variety of statistical time series to use as leading indicators for the epidemic waves in the UK during the pandemic between August 2020 and March 2022. We also compared the performance of these phylogeny-derived leading indicators with a range of non-phylogeny-derived leading indicators. Our experiments simulated data generation and real-time analysis.

**Findings:**

Using phylogenomic analysis, we identified leading indicators that would have generated EWS ahead of significant increases in COVID-19 hospitalisations in the UK between August 2020 and March 2022. Our results also show that EWS lead time is sensitive to the threshold set for the number of false positive (FP) EWS. It is often possible to generate longer EWS lead times if more FP EWS are tolerated. On the basis of maximising lead time and minimising the number of FP EWS, the best performing leading indicators that we identified, amongst a set of 1.4 million, were the maximum logistic growth rate (LGR) amongst clusters of the dominant Pango lineage and the mean simple LGR across a broader set of clusters. In the case of the former, the time between the EWS and wave inflection points (a conservative measure of wave start dates) for the seven waves ranged between a 20-day lead time and a 7-day lag, with a mean lead time of 5.4 days. The maximum number of FP EWS generated prior to a true positive (TP) EWS was two and this only occurred for two of the seven waves in the period. The mean simple LGR amongst a broader set of clusters also performed well in terms of lead time but with slightly more FP EWS.

**Interpretation:**

As a result of the significant surveillance effort during the pandemic, early detection of SARS-CoV-2 variants of concern Alpha, Delta, and Omicron provided some of the first examples where timely detection and characterisation of pathogen variants has been used to tailor public health response. The success of our method in generating early warning signals based on phylogenomic analysis for SARS-CoV-2 in the UK may make it a worthwhile addition to existing surveillance strategies. In addition, the method may be translatable to other countries and/or regions, and to other pathogens with large-scale and rapid genomic surveillance.

**Funding:**

This research was funded in whole, or in part, by the 10.13039/100010269Wellcome Trust (220885_Z_20_Z). For the purpose of open access, the author has applied a CC BY public copyright licence to any Author Accepted Manuscript version arising from this submission. KOD, OB, VBF and EMV acknowledge funding from the MRC Centre for Global Infectious Disease Analysis (reference MR/X020258/1), jointly funded by the 10.13039/501100000265UK Medical Research Council (MRC) and the UK Foreign, Commonwealth & Development Office (FCDO), under the 10.13039/501100000265MRC/10.13039/501100020171FCDO Concordat agreement and is also part of the EDCTP2 programme supported by the 10.13039/501100000780European Union. RMC acknowledges funding from the 10.13039/100010269Wellcome Trust Collaborators Award (206298/Z/17/Z).

**Research in context**

**Evidence before this study**

We searched PubMed on 29 June 2023 using the keywords ("COVID-19∗" OR "SARS-CoV-2∗") AND ("early?warning∗" OR "leading?indicator∗"). This returned 1013 articles. While we have not reviewed each of these articles, the set contains numerous studies of the SARS-CoV-2 genome and mutations with various analyses used to infer the impact on viral characteristics such as transmissibility and severity, and therefore provide an early warning. It also contains studies looking at producing early warning signals using non-phylogenomic leading indicators. By adding (“phylo∗”) as an additional search term, using the ‘AND’ Boolean operator, the number of articles was reduced to 21. This includes a study that demonstrated the use of counts of amino acid changes to detect the emergence of SARS-CoV-2 variants of interest/concern. However, our search did not identify any previous studies on the use of similar phylogenomic analysis as a method for generating early warning signals for epidemic waves of SARS-CoV-2 infections.

**Added value of this study**

We present a methodology for generating early warning signals of epidemic waves of COVID-19 infections in the UK, based on phylogenomic analysis of cluster logistic growth rates using geo-matched comparator sets. This has been made possible by the significantly higher number of SARS-CoV-2 genome sequences recorded during the COVID-19 pandemic relative to that in databases of other infectious disease pathogens. Using a relatively simple method for generating early warning signals, we have demonstrated that it would have been possible to produce lead times ahead of COVID-19 epidemic wave peaks that we judge to be useful for public health policymakers.

**Implications of all the available evidence**

The resulting lead times of the early warning signals generated suggest that the methodology may be useful if incorporated into broader surveillance programmes. There is also potential for future work to assess the performance of the methodology for other countries and/or regions, as well as other pathogens with large-scale and rapid genomic surveillance.

## Introduction

The coronavirus disease 2019 (COVID-19) pandemic has been typified by recurrent epidemic waves associated with distinct severe acute respiratory syndrome coronavirus 2 (SARS-CoV-2) variants. Early detection of epidemic waves can enable countermeasures to be implemented such as non-pharmaceutical interventions (social distancing), additional vaccination (booster campaigns), or healthcare capacity adjustments. In an article published in June 2022^1^ members of the World Health Organization's Technical Advisory Group on Virus Evolution (TAG-VE), tasked with implementing a global risk-monitoring framework for SARS-CoV-2 variants, and colleagues highlighted the importance of early warning systems and called for strengthened surveillance and the continued monitoring of SARS-CoV-2.

A variety of statistical methods have been developed, and/or borrowed from other scientific fields, with the objective of providing early warning signals (EWS) of the re-emergence of infectious diseases.^2^, ^3^, ^4^, ^5^ Often the leading indicator used in such methods is the incidence or prevalence of the infectious disease being monitored,^5^ with detrending and/or standardisation applied prior to statistical analysis.^6^ Machine learning has also been applied to the generation of EWS and has shown potential to increase the sensitivity and specificity of EWS.^7^

In addition to the generation of infectious disease EWS from the incidence and prevalence of the infectious disease itself, which we term ‘direct data’, researchers have sought to generate early warnings, and/or model the trajectory of the epidemic, using a range of potential leading indicators derived from ‘indirect data’ (i.e. data which does not directly measure the number of cases of infection). These include polymerase chain reaction (PCR) cycle threshold (Ct) levels from diagnostic tests,^8^^,^^9^ behavioural changes relating to social contact^10^ and mobility,^11^ wastewater analysis,^12^ internet search,^13^ social media usage,^13^ and work absenteeism.^14^ Counts of amino acid changes in virus samples^15^ and analysis of amino acid features to predict mutation spread^16^ have also been used to detect or forecast, respectively, the emergence of SARS-CoV-2 variants of interest/concern.

Because epidemic waves of SARS-CoV-2 infections are typically associated with particular variants,^17^ rapid detection of growing genomic variants can serve as a predictor of future waves. As a result of the significant surveillance effort during the pandemic, early detection of SARS-CoV-2 variants of concern Alpha, Delta, and Omicron provided some of the first examples in infectious disease epidemiology where the timely detection and characterisation of pathogen variants has been used to tailor public health response. The large amount of SARS-CoV-2 genomic sequence data produced during the pandemic^18^ has provided a unique opportunity to explore the possibility of using such data as the basis for generating an EWS for SARS-CoV-2 epidemic waves. The success of our method for SARS-CoV-2 in the UK may be indicative of what may be possible if genomic surveillance for other pathogens were to be massively increased.

We developed an analytical pipeline (Transmission Fitness Polymorphism Scanner)^17^^,^^19^ for systematically exploring all clades within a SARS-CoV-2 virus phylogeny to detect variants showing unusually high growth rates. We investigated the use of these cluster growth rates as the basis for a variety of statistical time series to use as leading indicators for the epidemic waves in the UK during the pandemic between August 2020 and March 2022. Our experiments simulated data generation and real-time analysis, and identified leading indicators that would have generated EWS ahead of significant increases in COVID-19 hospitalisations in the UK during this period. Other leading indicators generated using this method also compared favourably against a range of non-phylogenomic potential leading indicators with EWS generated using broadly the same method with some adjustments to make comparison possible.

## Methods

### Analysis of SARS-CoV-2 phylogenies using the Transmission Fitness Polymorphism (TFP) Scanner

**Fig. 1:**
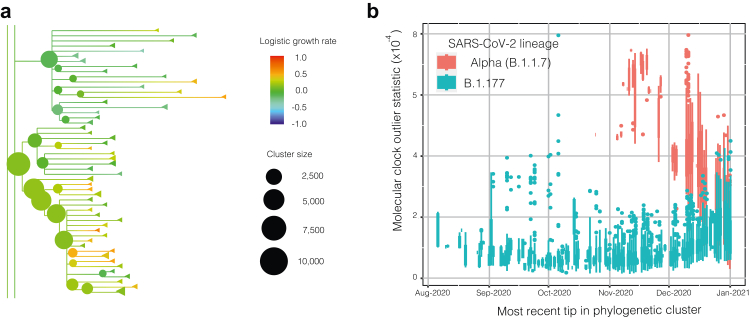
**Transmission Fitness Polymorphism (TFP) Scanner output example**: (a) extract from cluster phylogeny (tree date = 25 February 2021, minimum cluster age = 7 days, maximum cluster age = 56 days, minimum number of descendants = 20) showing the relative logistic growth rates between clusters and their size; (b) ‘molecular clock outlier’ (MCO) statistic boxplot showing elevated values as Alpha (B.1.1.7) replaced B.1.177 as the predominant variant in the UK.

We used the Transmission Fitness Polymorphism (TFP) Scanner^17^^,^^19^ to analyse a set of large SARS-CoV-2 phylogenies spanning August 2020 to March 2022. Whereas many statistics are generated by the TFP Scanner and many variations of this analysis are possible depending on cluster thresholds, we systematically explored a wide range of statistics and thresholds as the basis for EWS leading indicators. The analysis included the calculation of logistic growth rates (LGRs) for clusters (monophyletic clades above a given size threshold) within each phylogeny using two different methods: (1) a generalised linear model (GLM) to calculate the log odds of a sample being from a cluster of interest compared to a geographically (by country) and temporally matched sample weighted by prevalence, and multiplied by the estimated mean generation time of 6.5 days^20^, ^21^, ^22^, ^23^ to calculate the relative LGR per generation for each cluster of interest (the method is not sensitive to the value selected for generation time, see Supplementary Material for details); and (2) a generalised additive model (GAM) combined with a Gaussian process model to identify changes in growth rates over time. A third growth rate output is computed for each cluster as being either (1) or (2) depending on the level of model support calculated using the Akaike Information Criterion (AIC) and ‘relative likelihood’. Fig. 1a shows an example of the TFP Scanner output as viewed in the online html tree viewer.

The TFP Scanner also computes a ‘molecular clock outlier’ (MCO) statistic that measures the degree to which evolutionary rates differed in the lineage leading to a phylogenetic cluster (example shown in Fig. 1b). This statistic uses root-to-tip regression to predict the divergence of tips in a cluster and contrasts this with divergence within an ancestral clade including the given cluster. This predicted divergence is then compared to the true divergence of the cluster. If the predicted values based on the ancestral clade are very different from the observed values (p < 0.05) the cluster of interest is considered to be a MCO.

We used 24 different parameter sets in the TFP Scanner analysis, varying: the minimum cluster age ∈ {7, 14, 28 days}; the maximum cluster age ∈ {56, 84 days}; and the minimum threshold size for the number of descendants in clusters ∈ {20, 50, 100, percentage of genomic sequences within the maximum sample date period, such that the minimum number of descendants across the time series is 20 (0.32% for 56-day period and 0.24% for 84-day period)}.

### Calculation of early warning signals and ranking of leading indicators

We used the calculated cluster growth rates and MCO values, along with other statistics computed from the phylogeny, as the basis for 19 potential leading indicator types (listed in Supplementary Table S2 in the Supplementary Materials). We applied filters to the set of clusters to be included in the production of leading indicator time series. Only extant clusters (containing recent samples) were included and overlapping tips were removed to maintain independence of the cluster growth rates. Only external clusters (all descendants of the cluster most recent common ancestor (MRCA) included in the cluster) were included although this requirement was relaxed depending on the ratio of the growth rates between parent and sub-clusters in order to examine the inclusion of larger parent clusters where growth rates were similar to their sub-clusters. Where replacement was allowed, we varied the growth rate ratio between 60% and 100%. We also filtered clusters based on the p-value for the GLM calculated logistic growth rate ∈ {<0.01, <0.05 and no filter}. In total this added 30 unique filter sets to the analysis.

The time series were standardised using a ‘robust’ z-score (a.k.a. median absolute deviation method), with the resulting values compared against a range of thresholds on a chronological ‘add-one-in’ basis (simulating real-time analysis) to generate early warning signals (EWS). These indicators included realistic delays to simulate the time required to carry out genomic sequencing and execute bioinformatic pipelines (quality control and phylogenetic analysis). Positive EWS were classified as true or false according to the presence of the predominant SARS-CoV-2 variant during an epidemic wave being a significant contributor to the EWS generation. A variant was defined as being a significant contributor based on it being the most prevalent variant within a large proportion of phylogenetic clusters and/or the most prevalent variant in the phylogenetic clusters with the highest growth rates. Further details can be found in the Supplementary Material.

We calculated EWS lead times relative to COVID-19 epidemic wave start dates, which we defined by applying an optimised GAM to new hospital admissions data in the UK. These start dates are effectively the inflection points in between epidemic waves and represent a conservative date against which to measure the lead times of our EWS. Note that these inflection points would not be discernible from hospitalisation data in real time but are only apparent retrospectively.

The combination of TFP Scanner input parameter sets (24), the application of variable cluster filters (30), multiple potential leading indicators (19), and a range of EWS threshold levels (101), resulted in a set of 1.38 million unique EWS time series. The leading indicators were ranked on the basis of both EWS lead time and the number of false positives, with the aim of maximising lead time and minimising false positives.

Leading indicator parameter sets were filtered for those that had at least one true positive EWS per epidemic wave. We also applied filters limiting the number of false positive EWS per wave and ranking of leading indicators was based on lead time. Both criteria were applied across different combinations of waves. Further details of these criteria can be found in the Supplementary Material.

For comparison purposes, we also generated EWS from non-phylogeny-derived potential leading indicators (i.e. new hospital admissions, test positivity rates, PCR Ct levels, CoMix survey, Google mobility), using the same methodology format of time series standardisation and EWS generation above a threshold. Some adjustments to the methodology were made due to differences in the time range and data point frequency in the data sets. A different method of assessing performance was also required because it was not possible to differentiate between true and false positives in the same way for the non-phylogeny-derived leading indicators. A time window around the wave start (inflection) date or *R*_*t*_ critical transition date (when the time varying, or effective, reproduction number, or reproductive ratio, *R*_*t*_, increases above 1) was used to define the four elements of the confusion matrix. Further details of the methodology are described in the Supplementary Material.

### Data sources

We obtained a set of 288 SARS-CoV-2 phylogenetic trees used in our analysis from the Cloud Infrastructure for Microbial Bioinformatics (CLIMB).^24^ These were generated routinely and periodically between 14 August 2020 and 29 March 2022 using genomic sequence data from the COVID-19 Genomics UK (COG-UK) Consortium by the Phylopipe pipeline (https://github.com/virus-evolution/phylopipe). Trees were generated using maximum likelihood (ML) methods until March 2021, with later trees generated from a single ML tree by updating it using maximum parsimony methods. Contemporary trees were used in order to simulate real-time analysis and, in particular, to avoid including data that were subsequently revised. Genomic sequences in the trees were linked to patient case metadata, sourced from COG-UK via CLIMB on 3 May 2022. This enabled positive filtering for the genomic sequences collected in the UK under Pillar 2 (P2) sampling which was based primarily on community COVID-19 testing^25^ between April 2020 and the end of March 2022. Only P2 samples were selected to eliminate sampling bias present in Pillar 1 (P1) hospital samples, as well as to garner a more representative sample of transmission in the general population. Additional details can be found in the Supplementary Material.

### Role of the funding source

The funders of the study had no role in study design, data collection, data analysis, data interpretation, or writing of the report and the decision to submit the paper for publication.

## Results

**Fig. 2:**
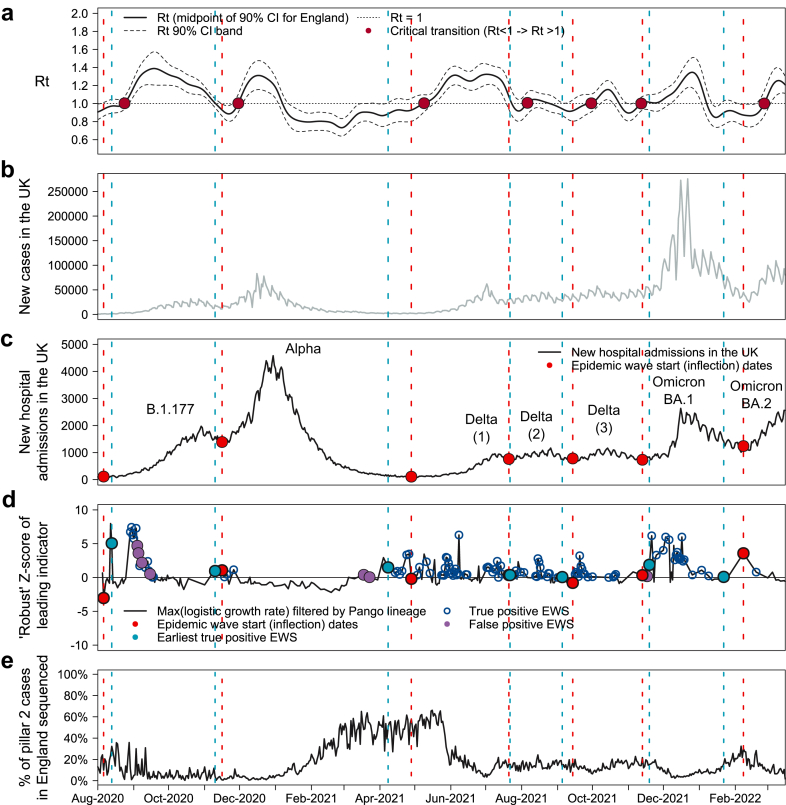
**Early warning signals for COVID-19 epidemic waves in the UK generated by analysis of SARS-CoV-2 phylogenetic trees.** (a) Estimated COVID-19 reproduction number (*R*_*t*_) with 90% confidence intervals and ‘critical transitions’ (when *R*_*t*_ increases above 1) marked. (b) New recorded positive cases of COVID-19 in the UK. (c) New COVID-19 hospital admissions in the UK with epidemic wave start (inflection) dates marked. (d) ‘Robust’ Z-score for one of the best performing leading indicators identified using our method. Earliest true positive (TP) EWSs (light blue) and wave start (inflection) points (red) marked. The mean earliest TP EWS lead time was 5.4 days. It ranged from a 20-day lead time to 7-day lag, but for all waves the earliest TP EWS was generated ahead of a significant rise in both hospitalisations and cases. (e) Estimate of SARS-CoV-2 sequencing ratio.

EWS performance varied across the potential leading indicators and the analysis variables investigated, which in combination represented 1.38 million unique parameter sets. Of these, 40,720 generated at least one true positive (TP) EWS for each of the seven epidemic waves during the period investigated. Fig. 2 shows an example of the EWS generated from one of the potential leading indicators we investigated. It is one of the highest ranked parameter sets on the basis of TP EWS lead-time and the number of false positive (FP) EWS generated.

Fig. 2 shows that the earliest TP EWS (d) provide a meaningful lead time ahead of the growth in new hospital admissions (c) across multiple waves. The mean lead time across all seven waves is 5.4 days for these parameters. The earliest lead time is 20 days, for the first Delta wave, and the latest lag time is 7 days, for the B.1.177 wave. Our leading indicator time series begins on 14 August 2020 and our computed start date for the B.1.177 wave is 19 August 2020, so given the limited number of data points in the leading indicator time series ahead of this wave, it is not surprising that the EWS lead time performance is weaker than for other waves. The earliest TP EWS generated for the B.1.177 wave by any of the parameters that produced at least one TP EWS for each wave is a 6-day lag. Our selected best leading indicator parameter set matches the earliest TP EWS amongst this group for three out of the seven waves and is within 4 days for all waves. This parameter set also performed well in terms of FP EWS, with just eight generated across all seven waves and only four before the earliest TP EWS, split across just two waves. Further details of the lead times for each wave are shown in Table 1.

**Table 1:** Early warning signals (EWS) generated by selected phylogeny-derived leading indicator for COVID-19 waves of infection in the UK.

	B.1.177	Alpha	Delta (1)	Delta (2)	Delta (3)	Omicron BA.1	Omicron BA.2
Epidemic wave start (inflection) date	19 Aug 2020	29 Nov 2020	11 May 2021	3 Aug 2021	27 Sep 2021	26 Nov 2021	21 Feb 2022
$R_{t}$ critical transition date	6 Sep 2020	13 Dec 2020	22 May 2021	19 Aug 2021	13 Oct 2021	25 Nov 2021	11 Mar 2022
Earliest true positive EWS date	26 Aug 2020	23 Nov 2020	21 Apr 2021	4 Aug 2021	18 Sep 2021	2 Dec 2021	4 Feb 2022
EWS lead time (days) relative to wave start (inflection) date Lead (−ve) and Lag (+ve)	+7	−6	−20	+1	−9	+6	−17
Number of false positives							
Prior to earliest true positive	0	0	2	0	0	2	0
After earliest true positive	4	0	0	0	0	0	0
Positive predictive value i.e. precision	0.76	1.00	0.95	1.00	1.00	0.90	1.00
Change in number of daily hospital admissions							
Between EWS and wave start (inflection) date							
Number	−17	−200	−64	−17	+23	−106	−178
%	−13%	−13%	−37%	−9%	+3%	−13%	−13%
Between EWS and wave peak							
Number	+1843	+2990	+2432	+1843	+406	+1795	+1263
%	+1418%	+188%	+1406%	+40%	+53%	+214%	+89%

The best leading indicator generated an earliest true positive (TP) EWS ranging from a 20-day lead time to a 7-day lag time, with a mean lead-time of 5.4 days across the seven epidemic waves. In all waves, the earliest TP EWS was ahead of significant increases in COVID-19 hospitalisations. A total of eight false positive (FP) EWS were generated, but only four of these were ahead of the earliest TP EWS and they only occurred in two of the seven waves. EWS information shown was generated by a leading indicator time series using the maximum logistic growth rate with a filter applied derived from the dominant Pango lineage (‘Dominant Pango lineage max LGR’, as described in the ‘Phylogeny-derived leading indicators investigated’ section in the Supplementary Material). The specific parameters used in the TFP Scanner to derive this leading indicator are shown in the first column of Table 2.

The leading indicator shown in Fig. 2d and Table 1 was derived by identifying the Pango lineage^26^ that was dominant (highest sample frequency within a cluster) in the most clusters and computing the maximum logistic growth rate (LGR) among the clusters where this Pango lineage was dominant. The specific parameters used in the generation of the leading indicator time series are shown in the first column of Table 2. These specific parameters were selected as the ‘best’ leading indicator in 9 of the 20 filtering and ranking criteria that produced results.

**Table 2:** Parameters for best performing phylogeny-derived leading indicators.

	Best performance on lead time and number of false positives	Best performance on lead time	Range of values investigated
Leading indicator	Dominant Pango lineage max LGR	Simple logistic growth rate (LGR) mean	See Supplementary Table S2 in Supplementary Materials
Transmission Fitness Polymorphism (TFP) Scanner parameter			
Minimum cluster age	7 days	14 days	7, 14, 28 days
Maximum cluster age	56 days	56 days	56, 84 days
Minimum number of descendants	20	20	20, 50, 100, % of samples
Cluster filter			
LGR p-value limit	≤0.01	No limit	≤0.01, ≤0.05, no limit
LGR threshold to determine replacement of sub-clusters with parent clusters	85%	85%	60%–100% with 5% increments, and no replacement
EWS generation			
‘Robust’ Z-score threshold for generating EWS	0.00	0.00	0.00–5.00 with 0.05 increments

Parameters used in Transmission Fitness Polymorphism (TFP) Scanner and subsequent variable cluster filters used in generating the two leading indicator time series selected as the best performing on the basis of a selection of ranking criteria. The dominant Pango lineage max logistic growth rate (LGR) leading indicator generated a mean earliest true positive (TP) early warning signal (EWS) lead time of 5.4 days across the seven epidemic waves (range from a 20-day lead time to a 7-day lag) and a total of eight false positive (FP) EWS were generated, but only four were ahead of the earliest TP EWS and they only occurred in two of the seven waves. This compares with the mean simple LGR leading indicator which had a mean earliest TP EWS of 6.4 days (range 24-day lead to 6-day lag) and generated 20 FP EWS, 16 of which were ahead of the earliest TP EWS. In all waves, the earliest TP EWS was ahead of significant increases in COVID-19 hospitalisations. More detailed Information on the EWS performance of the dominant Pango lineage max LGR leading indicator is shown in Table 1 and for other highly ranked leading indicators in Table 3.

We found that other leading indicators also performed well and by some measures performed better. Details of the best leading indicators for each set of filtering and ranking criteria are in Table 3. The leading indicator derived from the mean of the simple LGRs amongst phylogenetic clusters (specific parameters shown in the second column of Table 2) also performed well under the criteria with no restriction on the number of FP EWS. It produced similar lead times to those shown in Fig. 2 and Table 1 and the mean lead time across all seven waves was higher (6.4 days compared with 5.4 days for the dominant Pango lineage leading indicator), but with a higher level of mean FP EWS (2.9 vs 1.1).

**Table 3:** ‘Best’ parameter sets based on range of earliest true positive (TP) early warning signal (EWS) lead time and false positive (FP) EWS criteria.

	Best values by wave across all parameter sets	Best parameters for individual ranking and filter criteria																			
**Ranking criteria**																					
Rank by		Lead time	Lead time	Lead time	Lead time	Lead time	Lead time	Lead time	Lead time	Lead time	Lead time	Lead time	Lead time	Lead time	Lead time	Lead time	Lead time	Lead time	Lead time	Lead time	Lead time
Which waves included in ranking an filters		All	All	New variant driven	New variant driven	New variant driven	New variant driven	All	All	New variant driven	New variant driven	All	All	All	New variant driven	New variant driven	New variant driven	All	All	New variant driven	All
False positive limit		All	All	All	All	10	10	5	10	2	5	2	5	10	2	5	0	2	0	0	0
Which false positives		All	All	All	All	All	Before 1st TP	All	All	All	All	Before 1st TP	Before 1st TP	Before 1st TP	Before 1st TP	Before 1st TP	All	All	Before 1st TP	Before 1st TP	All
Restriction on individual wave lead times?		No	Yes	No	Yes	No	No	No	No	No	No	No	No	No	No	No	No	No	No	No	No
Number of parameter sets		40,720	19	40,720	19	12,066	13,554	2134	6998	2924	7164	1473	3143	10,313	3700	7642	995	319	608	1087	20
**TFP Scanner parameters**																					
Leading indicator		Mean simple LGR	Mean simple LGR	Mean LGR	Mean LGR	Mean GAM LGR	Mean GAM LGR	Dominant Pango lineage max LGR	Dominant Pango lineage max LGR	Dominant Pango lineage max LGR	Dominant Pango lineage max LGR	Dominant Pango lineage max LGR	Dominant Pango lineage max LGR	Dominant Pango lineage max LGR	Dominant Pango lineage max LGR	Dominant Pango lineage max LGR	Dominant Pango lineage max LGR	Dominant Pango lineage max LGR	Dominant Pango lineage max LGR	Dominant Pango lineage max LGR	Dominant Pango lineage max LGR
Cluster min age		14	14	7	7	14	14	7	7	7	7	7	7	7	7	7	7	14	14	14	28
Cluster max age		56	56	56	56	56	56	56	56	56	56	56	56	56	56	56	84	56	56	56	56
Cluster min descendants		20	20	20	20	20	20	20	20	20	20	20	20	20	20	20	20	20	20	20	20
**Cluster filter variables**																					
LGR p-value limit		No limit	No limit	0.01	0.01	0.01	0.01	0.01	0.01	0.01	0.01	0.01	0.01	0.01	0.01	0.01	0.01	0.05	0.05	0.05	0.01
Parent/sub-cluster replacement LGR threshold		85%	90%	60%	60%	80%	80%	85%	85%	85%	85%	85%	85%	85%	85%	85%	75%	85%	90%	90%	90%
EWS threshold		0.00	0.00	0.00	0.00	0.00	0.00	0.00	0.00	0.00	0.00	0.00	0.00	0.00	0.00	0.00	0.20	0.05	0.00	0.00	0.70
**Earliest TP EWS lead (−ve) or lag (+ve) days**																					
B.1.177	6	6	6	6	6	10	10	7	7	7	7	7	7	7	7	7	7	9	9	9	23
Alpha	−6	−6	−6	−6	−6	−6	−6	−6	−6	−6	−6	−6	−6	−6	−6	−6	−6	−6	−6	−6	−6
Delta (1)	−24	−24	−24	−24	−24	−24	−24	−20	−20	−20	−20	−20	−20	−20	−20	−20	−20	−20	−24	−24	−4
Delta (2)	−1	−1	−1	−1	−1	−1	−1	1	1	1	1	1	1	1	1	1	7	5	5	5	7
Delta (3)	−9	−9	−9	−1	−1	−1	−1	−9	−9	−9	−9	−9	−9	−9	−9	−9	0	2	2	2	2
Omicron BA.1	6	6	6	6	6	6	6	6	6	6	6	6	6	6	6	6	8	6	6	6	8
Omicron BA.2	−19	−17	−17	−19	−19	−19	−19	−17	−17	−17	−17	−17	−17	−17	−17	−17	−17	−17	−17	−17	11
Mean across all waves	−6.6	−6.4	−6.4	−5.6	−5.6	−5.0	−5.0	−5.4	−5.4	−5.4	−5.4	−5.4	−5.4	−5.4	−5.4	−5.4	−3.0	−3.0	−3.6	−3.6	5.9
Mean across variant driven waves	−10.8	−10.3	−10.3	−10.8	−10.8	−10.8	−10.8	−9.3	−9.3	−9.3	−9.3	−9.3	−9.3	−9.3	−9.3	−9.3	−8.8	−9.3	−10.3	−10.3	2.3
**Number of FP EWS**																					
B.1.177	0	5	5	1	1	5	5	4	4	4	4	4	4	4	4	4	5	2	4	4	0
Alpha	0	0	0	0	0	0	0	0	0	0	0	0	0	0	0	0	0	0	0	0	0
Delta (1)	0	14	14	17	17	6	6	2	2	2	2	2	2	2	2	2	0	0	0	0	0
Delta (2)	0	0	0	0	0	0	0	0	0	0	0	0	0	0	0	0	0	0	0	0	0
Delta (3)	0	0	0	0	0	0	0	0	0	0	0	0	0	0	0	0	0	0	0	0	0
Omicron BA.1	0	1	1	1	1	2	2	2	2	2	2	2	2	2	2	2	0	2	2	2	0
Omicron BA.2	0	0	0	3	3	3	3	0	0	0	0	0	0	0	0	0	0	0	0	0	0
Mean across all waves	0.0	2.9	2.9	3.1	3.1	2.3	2.3	1.1	1.1	1.1	1.1	1.1	1.1	1.1	1.1	1.1	0.7	0.6	0.9	0.9	0.0
Mean across variant driven waves	0.0	3.8	3.8	5.3	5.3	2.8	2.8	1.0	1.0	1.0	1.0	1.0	1.0	1.0	1.0	1.0	0.0	0.5	0.5	0.5	0.0
**Number of FP EWS before earliest TP**																					
B.1.177	0	1	1	0	0	3	3	0	0	0	0	0	0	0	0	0	0	0	0	0	0
Alpha	0	0	0	0	0	0	0	0	0	0	0	0	0	0	0	0	0	0	0	0	0
Delta (1)	0	14	14	14	14	6	6	2	2	2	2	2	2	2	2	2	0	0	0	0	0
Delta (2)	0	0	0	0	0	0	0	0	0	0	0	0	0	0	0	0	0	0	0	0	0
Delta (3)	0	0	0	0	0	0	0	0	0	0	0	0	0	0	0	0	0	0	0	0	0
Omicron BA.1	0	1	1	0	0	2	2	2	2	2	2	2	2	2	2	2	0	0	0	0	0
Omicron BA.2	0	0	0	1	1	1	1	0	0	0	0	0	0	0	0	0	0	0	0	0	0
Mean across all waves	0.0	2.3	2.3	2.1	2.1	1.7	1.7	0.6	0.6	0.6	0.6	0.6	0.6	0.6	0.6	0.6	0.0	0.0	0.0	0.0	0.0
Mean across variant driven waves	0.0	3.8	3.8	3.8	3.8	2.3	2.3	1.0	1.0	1.0	1.0	1.0	1.0	1.0	1.0	1.0	0.0	0.0	0.0	0.0	0.0
**Precision or positive predictive value (PPV)**																					
B.1.177	1.0	0.6	0.6	0.8	0.8	0.6	0.6	0.8	0.8	0.8	0.8	0.8	0.8	0.8	0.8	0.8	0.7	0.8	0.7	0.7	1.0
Alpha	1.0	1.0	1.0	1.0	1.0	1.0	1.0	1.0	1.0	1.0	1.0	1.0	1.0	1.0	1.0	1.0	1.0	1.0	1.0	1.0	1.0
Delta (1)	1.0	0.7	0.7	0.5	0.5	0.6	0.6	1.0	1.0	1.0	1.0	1.0	1.0	1.0	1.0	1.0	1.0	1.0	1.0	1.0	1.0
Delta (2)	1.0	1.0	1.0	1.0	1.0	1.0	1.0	1.0	1.0	1.0	1.0	1.0	1.0	1.0	1.0	1.0	1.0	1.0	1.0	1.0	1.0
Delta (3)	1.0	1.0	1.0	1.0	1.0	1.0	1.0	1.0	1.0	1.0	1.0	1.0	1.0	1.0	1.0	1.0	1.0	1.0	1.0	1.0	1.0
Omicron BA.1	1.0	0.8	0.9	0.8	0.8	0.8	0.8	0.9	0.9	0.9	0.9	0.9	0.9	0.9	0.9	0.9	1.0	0.9	0.9	0.9	1.0
Omicron BA.2	1.0	1.0	1.0	0.7	0.7	0.3	0.3	1.0	1.0	1.0	1.0	1.0	1.0	1.0	1.0	1.0	1.0	1.0	1.0	1.0	1.0
Mean across all waves	1.0	0.9	0.9	0.8	0.8	0.7	0.7	0.9	0.9	0.9	0.9	0.9	0.9	0.9	0.9	0.9	1.0	1.0	0.9	0.9	1.0
Mean across variant driven waves	1.0	0.9	0.9	0.8	0.8	0.7	0.7	1.0	1.0	1.0	1.0	1.0	1.0	1.0	1.0	1.0	1.0	1.0	1.0	1.0	1.0

Rankings are determined by the sum of the earliest TP EWS lead times across different sets of SARS-CoV-2 epidemic waves and a range of filters based on the number of FP EWS. There is some subjectivity in selecting a single parameter set for each ranking criteria as more than one parameter set may achieve the same ranking score. Tables containing the highest ranked parameter sets for each of the criteria sets in this table can be found in the Supplementary Material.

**Fig. 3:**
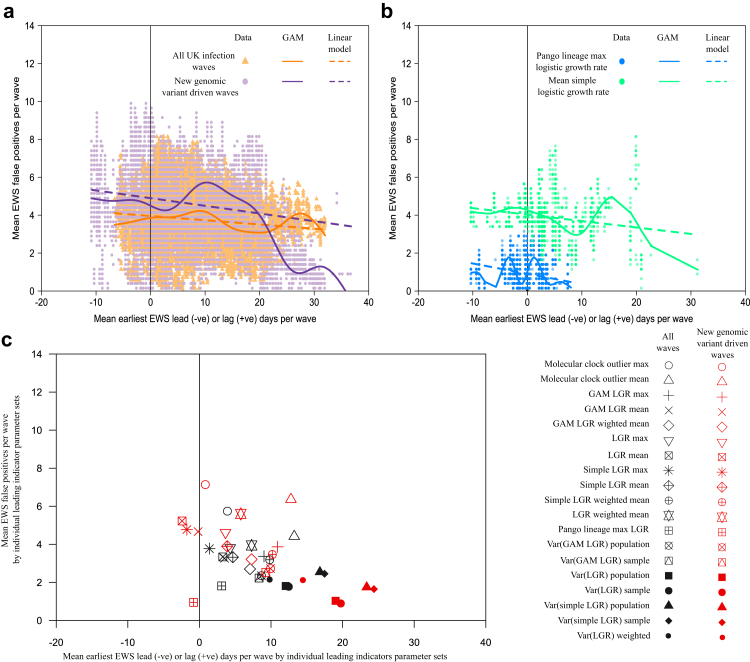
**Link between early warning signal (EWS) lead time and number of false positive (FP) EWS.** (a) Mean number of FP EWS against mean earliest true positive (TP) EWS date for full range of parameter sets. Mean calculated using all UK SARS-CoV-2 infection waves and, separately, the waves driven by new genomic variants: Alpha, Delta (1st wave), Omicron BA.1 & BA.2. (b) Repeat of (A) but only for two leading indicators (dominant Pango lineage max logistic growth rate (LGR) and mean Simple LGR–see Supplementary Material for full definitions) and only for the waves driven by new genomic variants. (c) Mean EWS results for each phylogenomic-derived leading indicator type.

The difference between the mean lead time and number of false positives for these two parameter sets also shows that in general, parameter sets that generate earlier TP EWS do so with a larger number of FP EWS. This is true for the majority (14 out of 19) of leading indicator types that we investigated, although not all. This relationship is further illustrated across all leading indicator parameter sets in Fig. 3a and b, which focuses on the two leading indicator types generating the best performing parameter sets.

The parameter sets selected as the best performing under the various filtering and ranking criteria are shown in Table 3. Due to multiple parameter sets sharing the same top score on total lead times across waves, there is some subjectivity in selecting the best performing parameter sets. Where parameter sets were tied on total EWS lead time, we considered the number of false positives as well as lead time performance for individual waves. The highest ranked parameter sets for each of the 20 unique ranking and filtering criteria sets that produced results are available in a separate file (SM1–Best EWS Results by Filter and Ranking Criteria) as part of the Supplementary Material.

Using a different method of assessing performance, which enabled comparison between the phylogeny-derived leading indicators and the non-phylogeny-derived leading indicators, we found that the best of the former outperformed the best of the latter. The highest ranked for each category are shown in Supplementary Table S7 in the Supplementary Material. The best performer in the former category was the mean cluster LGR, which had a minimum normalised Matthews Correlation Coefficient (MCC) across five epidemic waves (Alpha, Delta (1,2,3), Omicron BA.1) of 0.63 (range 0.63–0.93) and an arithmetic mean normalised MCC of 0.74. This compares with the minimum normalised MCC of 0.56 (range 0.56–0.68) and arithmetic mean normalised MCC of 0.63 for the best performer, Google mobility grocery & pharmacy, from the latter category. Time series plots for both are shown in Supplementary Figure S1 in the Supplementary Material.

We compared EWS results for the best performing phylogeny-derived leading indicator and parameter sets, shown in Table 2, generated using cluster matching at two geographic aggregations: country level, and the finer administrative level 2 (adm2) scale. EWS lead times were shorter (and lag times longer), and the number of false positives was higher at the finer scale geographic aggregation (see Supplementary Material for details), indicating that adm2 is too fine in this case.

## Discussion

We have demonstrated that this method for analysing SARS-CoV-2 phylogenomic data and extracting statistics would have produced early warning signals (EWS) for COVID-19 epidemic waves of hospital admissions in the UK.

One of the best leading indicators we found is the largest cluster logistic growth rate (LGR) amongst the dominant Pango lineage. This generated EWS ranging from a lead time of 20 days to a lag time of 7 days (see Table 1), with a mean lead time of 5.4 days. The lead time is dependent on our definition of the wave start date, which is conservative as it represents the inflection point between two wave peaks. However, this conservatism is offset to some extent by the use of hospitalisations rather than cases, which occur earlier but are less consistently measured. However, it can be seen in Fig. 2 that the earliest true positive (TP) EWSs also occur prior to significant increases in reported cases.

Leading indicators based on Pango lineages implicitly incorporate expert assessment of the impact of genomic mutations on the virus variant’s potential to infect humans. It is therefore perhaps unsurprising, and reassuring, that it ranks amongst the best leading indicators. While our analysis is retrospective, we simulated real-time analysis by only incorporating data available at each step in the time series. The labour intensive process of Pango lineage assignment, upon which this particular leading indicator relies, is unlikely to continue indefinitely. In any case, other leading indicator types that are not dependent on prior variant classification, such as the mean cluster simple LGR, also produced similar, and sometimes better, lead times, albeit with higher levels of false positive (FP) EWS. Encouragingly, the best leading indicators when ranked on performance across multiple waves also compare well to the best performance achieved when ranked by individual waves.

Our results show that EWS lead time can be sensitive to the threshold set for the number of FP EWS. For the majority of leading indicator types investigated, it is possible to generate longer EWS lead times if more FP EWS are tolerated. While FP EWS are undesirable and should ideally be minimised, we envisage the role of our EWS generation method as being an intermediate stage in surveillance strategy rather than a final determinant in policy decisions. Identification of fast-growing pathogen variants should serve as a prompt for in-depth analysis of the epidemiological and genomic characteristics of the clusters driving the generation of the EWS. In such a surveillance strategy workflow, health agencies place greater importance on lead time than the number of FP EWS. This may mean that leading indicator types and parameter sets other than those highlighted in our results may be more practically useful. However, the balance we struck between the two factors (lead time and FP EWS) resulted in lead times that were within 4 days of the best lead time amongst parameter sets that generated at least one TP EWS for all seven epidemic waves investigated, regardless of the number of FP EWS. Therefore, the improvement in lead times by allowing a larger number of false positives may be relatively limited. However, the filtering and ranking criteria we used are not exhaustive and so there may be other leading indicators and parameter sets within the 1.38 million produced that generate better EWS results.

O’Brien & Clements^27^ showed that the reliability of leading indicators, supported by critical-slowing-down theory,^28^^,^^29^ varies with COVID-19 wave. Dablander et al.^30^ also found that such leading indicators failed to identify the second COVID-19 wave in Europe, which they posit is due to the violation of the key assumption that there is a separation in the timescales such that the dynamics of the epidemic settle down to a quasi-equilibrium from which there is a slow drift towards the critical point, i.e. *R*_*t*_ = 1. We also saw variability in EWS performance across waves in our parameter sets with the earliest TP EWS ranging from a lead time of 20 days (for the first Delta wave) to a lag time of 7 days (for B.1.177) (see Table 1). However, the leading indicators that we investigated do not rely on critical-slowing-down theory and the mechanisms driving this variability are different. The performance of genomic EWS will also depend sensitively on sequence sampling activity which was highly variable over the course of the pandemic. There is also some indication that our method is more successful for waves that are primarily driven by new genomic variants (Alpha, first Delta and Omicron BA.2 EWS lead times range from 6 to 20 days) compared with waves likely resulting from a resurgence of existing genomic variants (1-day lag and 9-day lead time for second and third Delta waves respectively) due to factors such as varying levels of non-pharmaceutical interventions (NPIs). However, this conclusion requires the exclusion of two waves driven by new genomic variants: 1) B.1.177 (7-day lag time), for which we have limited data ahead of the wave start date and furthermore a substantial difference in transmissibility for this lineage is doubtful^31^; and 2) Omicron BA.1 (6-day lag time), which produced a much more rapid increase in cases and hospitalisations than other variants. Furthermore, the difference in transmissibility of BA.1 was abundantly clear from international data long before the wave began in the UK, which was not considered in our analysis, but arguably comprises a clear genomics-based EWS. While the EWS lead time differential between these two types of wave is not definitive, we would expect there to be a difference given that the EWS are derived from relative growth rates. These should be more pronounced when there is a new genomic variant outcompeting an existing predominant variant due to a higher level of transmissibility. When a genomic variant is already predominant amongst the prevailing infections, there is less likely to be a difference in growth rates of comparable localised outbreak clusters. Therefore the EWS generated by our methodology will be weaker and delayed, leading to shorter lead times or longer lag times. Further work could be undertaken to incorporate NPIs into the methodology.

It has been suggested that localised minor outbreaks are often seen in the period after a critical transition, i.e. when the time-varying reproduction number (*R*_*t*_) moves from <1 to >1, and prior to an epidemic wave.^32^ Our methodology is driven by the rapid identification of SARS-CoV-2 phylogenetic clusters with a growth advantage over other contemporary clusters. These clusters are by definition small relative to the size of an epidemic wave and we believe the leading indicators we investigated are detecting these localised minor outbreaks that are indicative of a critical transition of *R*_*t*_ ahead of the epidemic wave. However, for a majority of waves the EWS generated not only precede the wave start (inflection) dates, but also the estimated critical transition points for *R*_*t*_ (see Fig. 2 and Table 1). The rise in hospitalisations before the critical transition in *R*_*t*_ at first appears counterintuitive. However, the *R*_*t*_ estimate shown in Fig. 2a results from an aggregation of infections caused by all SARS-CoV-2 variants in circulation at the time and at the national level. It has been shown that different variants have different levels of transmissibility^33^ and so it is possible for some variants to have an *R*_*t*_ > 1 in geographically localised clusters while the value aggregated over the UK population is less than 1. Our interpretation is therefore that the EWS are being generated by localised clusters that have an *R*_*t*_ > 1 and that this precedes the transition of the estimated *R*_*t*_ above 1 for the population as a whole.

Following the emergence of Omicron BA.1, lineage dynamics have become more complex and co-circulation of multiple lineages with a growth advantage has become more common-place. The scanning methodology should be robust to identification of EWS from multiple co-circulating lineages. Growth statistics of a given clade of virus are measured relative to all co-circulating lineages, and do not presume a single incumbent reference lineage. Provided that multiple lineages are not emerging within exactly the same geographic area at the same time, they should trigger distinct EWS, although the robustness of this approach may be sensitive to the scale of geographic aggregation being used. The optimum geographic scale for use in cluster matching within the Transmission Fitness Polymorphism (TFP) Scanner remains an open question, and depends on stochastic epidemiological dynamics and correlated sampling.

SARS-CoV-2 testing and genomic sequencing policies in the UK varied during the period investigated^25^ and sample density in the UK has subsequently been significantly reduced. We expect that our EWS generation method is sensitive to sample density changes. Further modelling and theoretical analysis is needed to evaluate this sensitivity. This could include down-sampling of existing data and/or filtering for only clinical samples, to replicate the current sequencing policies in the UK, and observing the impact on EWS lead times. Once a greater understanding of this relationship has been established, the method could also be applied to other countries with sequencing capacity and sampling policies that differ from those in the UK.

Many countries are increasingly using wastewater surveillance as part of their epidemic preparedness strategies given the low cost and relative ease in sample collection across populations.^34^ Further work could examine the performance of EWS generated using a subset of SARS-CoV-2 sequences collected from wastewater samples compared with EWS generated from sequences originating from diagnostic test samples.

The best phylogeny-derived leading indicators compared favourably against the highest ranked leading indicators from a range of non-phylogeny-derived time series (listed in Supplementary Table S6 in the Supplementary Material). However, to make comparison of performance between phylogeny-derived and non-phylogeny-derived leading indicators possible it was necessary to adopt a different methodology. The precise parameters for the strongest performing phylogeny-derived leading indicators were different under the two methodologies, although broadly the same leading indicator types performed well under both. It should be noted that our focus has been on developing a method that generates EWS from leading indicators derived from the SARS-CoV-2 genome using the TFP Scanner. We have therefore not optimised the method for the non-TFP Scanner datasets and so these data sets may generate better (or worse) EWS using other methods.

In conclusion, we have demonstrated the ability to generate early warning signals (EWS) for epidemic waves of SARS-CoV-2 in the UK using leading indicators derived from the analysis of phylogenetic trees, and more fundamentally the analysis of pathogen genomes. The best performing leading indicator in terms of lead time and number of false positive EWS was the maximum logistic growth rate amongst phylogenetic clusters after filtering for the dominant Pango lineage. Other leading indicators that did not require the prior assignment of Pango lineage also performed similarly well in terms of lead time albeit with a greater number of false positive EWS. In our view, this method for generating EWS shows potential to be incorporated into surveillance strategy, in particular as a prompt for further genomic analysis, but given the reduction in testing and sequencing since the end of the period investigated, further work is required to determine the sensitivity of the method to the sampling frame (clinical or community sources) and sample size.

## Contributors

EV conceived this study and oversaw the analysis. EV and KD contributed to the study design. KD performed the analysis and led the writing of the paper. EV and OB developed the Transmission Fitness Polymorphism (TFP) Scanner that was used in the analysis. All authors contributed to the review and editing of the manuscript. KD and EV accessed and verified the data, and no authors were precluded from accessing the data. All authors share responsibility for the final decision to submit for publication. All authors have read and approved the final version of the manuscript.

## Data sharing statement

All code and data used in this study is publicly available online. The Transmission Fitness Polymorphism Scanner R package can be found here https://github.com/mrc-ide/tfpscanner. The additional code used in the analysis presented in this article can be found here https://github.com/KieranODrake/Early_Warning_Signal.

## Declaration of interests

All authors declare no competing interests.

## Appendix A Supplementary data

Supplementary Material

SM2–TFPS_vs_nonTFPS_Perc_Rank_Mean_Min_MCC

SM1–Best EWS Results by Filter and Ranking Criteria

## References

[1] Subissi L., von Gottberg A., Thukral L. (2022). An early warning system for emerging SARS-CoV-2 variants. Nat Med.

[2] Farrington C.P., Andrews N.J., Beale A.D., Catchpole M.A. (1996). A statistical algorithm for the early detection of outbreaks of infectious disease. J R Stat Soc A.

[3] Wagner M.M., Tsui F.-C., Espino J.U. (2001). The emerging science of very early detection of disease outbreaks. J Public Health Manag Pract.

[4] Unkel S., Farrington C.P., Garthwaite P.H., Robertson C., Andrews N. (2012). Statistical methods for the prospective detection of infectious disease outbreaks: a review. J R Stat Soc A.

[5] Southall E., Brett T.S., Tildesley M.J., Dyson L. (2021). Early warning signals of infectious disease transitions: a review. J R Soc Interface.

[6] Proverbio D., Kemp F., Magni S., Gonçalves J. (2022). Performance of early warning signals for disease re-emergence: a case study on COVID-19 data. PLoS Comput Biol.

[7] Bury T.M., Sujith R.I., Pavithran I. (2021). Deep learning for early warning signals of tipping points. Proc Natl Acad Sci USA.

[8] Hay J.A., Kennedy-Shaffer L., Kanjilal S. (2021). Estimating epidemiologic dynamics from cross-sectional viral load distributions. Science.

[9] Lin Y., Yang B., Cobey S. (2022). Incorporating temporal distribution of population-level viral load enables real-time estimation of COVID-19 transmission. Nat Commun.

[10] Jarvis C.I., van Zandvoort K., Gimma A. (2020). Quantifying the impact of physical distance measures on the transmission of COVID-19 in the UK. BMC Med.

[11] Kogan N.E., Clemente L., Liautaud P. (2021). An early warning approach to monitor COVID-19 activity with multiple digital traces in near real time. Sci Adv.

[12] SciLifeLab COVID-19 data portal Sweden - wastewater-based epidemiology in Sweden. https://www.covid19dataportal.se/dashboards/wastewater/.

[13] O’Leary D.E., Storey V.C. (2020). A Google–Wikipedia–Twitter model as a leading indicator of the numbers of coronavirus deaths. Intell Syst Account Financ Manag.

[14] Quenel P., Dab W., Hannoun C., Cohe J.M. (1994). Sensitivity, specificity and predictive values of health service based indicators for the surveillance of influenza A epidemics. Int J Epidemiol.

[15] Bernasconi A., Mari L., Casagrandi R., Ceri S. (2021). Data-driven analysis of amino acid change dynamics timely reveals SARS-CoV-2 variant emergence. Sci Rep.

[16] Maher M.C., Bartha I., Weaver S. (2022). Predicting the mutational drivers of future SARS-CoV-2 variants of concern. Sci Transl Med.

[17] Volz E.M. (2023). Fitness, growth and transmissibility of SARS-CoV-2 genetic variants. Nat Rev Genet.

[18] Stockdale J.E., Liu P., Colijn C. (2022). The potential of genomics for infectious disease forecasting. Nat Microbiol.

[19] Volz E.M., Boyd O. Transmission fitness polymorphism scanner. https://github.com/mrc-ide/tfpscanner.

[20] Bi Q., Wu Y., Mei S. (2020). Epidemiology and transmission of COVID-19 in 391 cases and 1286 of their close contacts in Shenzhen, China: a retrospective cohort study. Lancet Infect Dis.

[21] Flaxman S., Mishra S., Gandy A. (2020). Estimating the effects of non-pharmaceutical interventions on COVID-19 in Europe. Nature.

[22] Manica M., de Bellis A., Guzzetta G. (2022). Intrinsic generation time of the SARS-CoV-2 Omicron variant: an observational study of household transmission. Lancet Reg Health Eur.

[23] Qin W., Sun J., Xu P. (2020). The descriptive epidemiology of coronavirus disease 2019 during the epidemic period in Lu'an, China: achieving limited community transmission using proactive response strategies. Epidemiol Infect.

[24] Nicholls S.M., Poplawski R., Bull M.J. (2021). CLIMB-COVID: continuous integration supporting decentralised sequencing for SARS-CoV-2 genomic surveillance. Genome Biol.

[25] UK HM Government Department of Health & Social Care Policy paper: coronavirus (COVID-19): scaling up our testing programmes. https://www.gov.uk/government/publications/coronavirus-covid-19-scaling-up-testing-programmes/coronavirus-covid-19-scaling-up-our-testing-programmes.

[26] Rambaut A., Holmes E.C., O’Toole Á. (2020). A dynamic nomenclature proposal for SARS-CoV-2 lineages to assist genomic epidemiology. Nat Microbiol.

[27] O’Brien D.A., Clements C.F. (2021). Early warning signal reliability varies with COVID-19 waves. Biol Lett.

[28] Wissel C. (1984). A universal law of the characteristic return time near thresholds. Oecologia.

[29] Scheffer M., Bascompte J., Brock W.A. (2009). Early-warning signals for critical transitions. Nature.

[30] Dablander F., Heesterbeek H., Borsboom D., Drake J.M. (2022). Overlapping timescales obscure early warning signals of the second COVID-19 wave. Proc R Soc B.

[31] Hodcroft E.B., Zuber M., Nadeau S. (2021). Spread of a SARS-CoV-2 variant through Europe in the summer of 2020. Nature.

[32] Dibble C.J., O’Dea E.B., Park A.W., Drake J.M. (2016). Waiting time to infectious disease emergence. J R Soc Interface.

[33] Volz E.M., Mishra S., Chand M. (2021). Assessing transmissibility of SARS-CoV-2 lineage B.1.1.7 in England. Nature.

[34] Ahmed W., Simpson S.L., Bertsch P.M. (2022). Minimizing errors in RT-PCR detection and quantification of SARS-CoV-2 RNA for wastewater surveillance. Sci Total Environ.

