# Supplementary material for: Phylogenomic early warning signals for SARS-CoV-2 epidemic waves
Source: eBioMedicine. 2024 Jan 8;100:104939. doi: 10.1016/j.ebiom.2023.104939 (PMC10792554; doi:10.1016/j.ebiom.2023.104939)
Supplement: Supplementary Material [file mmc1.pdf]

Phylogenomic early warning signals for SARS-CoV-2 epidemic waves

Supplementary Material

Table of Contents

Methods 1
Overview 1
SARS-CoV-2 data 2
Transmission Fitness Polymorphism (TFP) Scanner 2
Table S1: Input parameters for TFP Scanner R package 4
Phylogeny-derived leading indicators investigated 5
Table S2: Summary of potential leading indicators derived from TFP Scanner outputs 6
Calculation of early warning signals (EWS) 6
Table S3: COVID-19 epidemic wave start (inflection) dates in the UK 8
Table S4: EWS generated by selected phylogeny-derived leading indicators 10
Assessment of leading indicators 9
Table S5: Filters and ranking criteria applied to EWS results from parameter sets 11
Comparison between phylogeny- and non-phylogeny-derived leading indicators 11
Table S6: Non-phylogeny-derived leading indicators investigated 13
Figure S1: EWS for COVID-19 epidemic waves in the UK assessed using 'time window' method 16
Table S7: Highest ranked genomic- and non-genomic-derived leading indicators 17
Analysis pipeline 16
Sensitivity Analyses 18
Generation time 18
Figure S2: Methodology is not sensitive to value used for generation time 18
Geographic aggregation 18
Table S8: Strongest performing leading indicators using country level geographic aggregation for cluster matching, show weaker performance with finer geographic aggregation 19
Supplementary Material References 20

Methods

Overview

We used the Transmission Fitness Polymorphism (TFP) Scanner[1,2] (designated in an R package mrc-ide/tfpscanner) to analyse a set of large severe acute respiratory syndrome coronavirus 2 (SARS-CoV-2) phylogenies spanning August 2020 to March 2022. The analysis included the calculation of growth rates for clusters, or clades, within each phylogeny. These growth rates were used, along with other statistics computed from the phylogeny, as the basis for a variety of potential leading indicator time series. The time series were standardised using a 'robust' z-score, with the resulting values compared against a range of thresholds on a chronological 'add-one-in' basis (simulating real-time analysis) to generate early warning signals (EWS). Positive EWS were categorised as true or false and the leading indicators were ranked on the basis of both EWS

lead time and the number of false positives. A variety of parameter sets were used in the analysis, resulting in a set of 1·38 million early warning signal time series.

SARS-CoV-2 data

We obtained a set of 288 SARS-CoV-2 phylogenetic trees used in our analysis from the Cloud Infrastructure for Microbial Bioinformatics (CLIMB).[3] These were generated routinely and periodically between 14 August 2020 and 29 March 2022 using genomic sequence data from the COVID-19 Genomics UK (COG-UK) Consortium by the Phylopipe pipeline (https://github.com/virus-evolution/phylopipe). Trees were generated using maximum likelihood (ML) methods until March 2021, with later trees generated from a single ML tree by updating it using maximum parsimony methods. Contemporary trees were used in order to simulate real-time analysis and, in particular, to avoid including data that were subsequently revised. Genomic sequences in the trees were linked to patient case metadata, sourced from COG-UK via CLIMB on 3 May 2022. This enabled positive filtering for the genomic sequences collected in the UK under Pillar 2 (P2) community samples[4] between April 2020 and the end of March 2022. Only P2 samples were selected to eliminate sampling bias present in Pillar 1 (P1) hospital samples, as well as to garner a more representative sample of transmission in the general population. Genomic sequences without associated dates or with erroneous dates were removed.

Transmission Fitness Polymorphism (TFP) Scanner

The TFP Scanner[1,2] was used to calculate logistic growth rates (LGRs) for clusters (or clades) within each SARS-CoV-2 phylogenetic tree. Clusters are defined as including all the descendants from a given node in the tree. Comparison samples for statistical analyses are selected as ancestral nodes including all their descendants, which exclude the present cluster. The growth rates are estimated relative to a comparator set of geographically- (by country) and temporally-matched lineages weighted by prevalence. This controls for bias that accrues from many unmeasured confounders that may amplify transmission in a certain time or place. For evolutionary statistics, a comparator is selected based on shared ancestry.

The TFP Scanner incorporates multiple statistical methods into one function for identifying emerging variants or clusters of interest or concern, as well as to conduct real-time monitoring of outbreak growth over time. It is designated in an R package, *mrc-ide/tfpscanner*, which includes an online html tree viewer for the whole tree output (*example of a section of such a tree output is shown in Figure 1a in the main article*). The TFP Scanner also outputs a data frame in which each row represents a cluster with associated cluster metadata including: most recent sample date; least recent sample date; cluster size; LGR (computed as either the simple LGR or the generalised additive model (GAM) LGR depending on the level of model support determined using the Akaike Information Criterion (AIC) and the 'relative likelihood'); simple LGR; GAM LGR; simple logistic model support; clock outlier statistic; lineages; co-circulating lineages; region summary; defining mutations; and all mutations within cluster.

Three statistical methods for monitoring growth are used by the TFP Scanner:

1. A generalised linear model (GLM) is used to calculate the logistic odds of a sample being from a cluster of interest compared to a matched sample over time. The logistic odds of being a cluster of interest is multiplied by the estimated mean generation time (6·5 days assumed for SARS-CoV-2[5-8]) to calculate the relative growth rate per generation time period for each cluster of interest. The associated p-value is also recorded.
2. A generalised additive model (GAM) combined with a Gaussian process model to identify changes in growth over time.
3. A GAM combined with a model of spatial correlation between neighbouring lower-tier local authorities (LTLAs) (or other specified administration level), using a Gaussian Markov random model, to smooth estimates over sparse observations. Note that this third method was not used in the investigation presented in this article.

The TFP Scanner also computes a 'molecular clock outlier' (MCO) statistic that measures the degree to which evolutionary rates differed in the lineage leading to a clade (*an example is shown in Figure 1b in the main article*). This statistic uses root-to-tip regression to predict the divergence of tips in a cluster based on an ancestral clade. This predicted divergence is then compared to the true divergence of the cluster. If the predicted values, based on the ancestral clade, are very different from the observed values ($p < 0·05$) the cluster of interest is considered a 'clock outlier'.

The majority of inputs to the TFP Scanner function were kept constant, but some parameters were varied as part of our search for the best leading indicators derived from the TFP Scanner outputs:

- The minimum threshold size for the number of descendants in clusters was varied ∈ {20, 50, 100, percentage of genomic sequences within the maximum sample date period, such that the minimum number of descendants across the time series is 20 (0·32% for 56-day period and 0·24% for 84-day period)} while the maximum was held constant at 20,000.
- Minimum cluster age ∈ {7, 14, 28 days}
- Maximum cluster age ∈ {56, 84 days}

Table S1 shows the full set of parameters used in the TFP Scanner runs.

The time taken for genomic sequences from samples to be processed and incorporated into a published phylogenetic tree varied over the period that we investigated. After removing 16 samples that had dates later than the tree date in which they first appeared, the difference between the most recent UK Pillar 2 sample in a tree and the date of publication for the tree was as much as 23 days and as low as 0 days. It seems implausible that a sample was sequenced and incorporated into a phylogenetic tree on the same day as the diagnostic test. To account for this we identified the earliest set of dates that would include 99·9% of samples for each tree. Any samples with dates falling outside of this range were removed from all trees prior to analysis. This may also have the effect of reducing sampling bias due to faster sample processing at some laboratories, which would otherwise be over-represented.

There is also the possibility of misdated samples with an erroneous earlier date (such as https://github.com/COG-UK/dipi-group/issues/175), which we would not identify or correct with our current method.

In this article, we took a historical record approach, using the date of production of the phylogenetic trees as the dates for our leading indicator time series. We also considered a second approach, although the results are not reported in this article. This alternative approach would take into account improvements that were made in the pipeline from diagnostic test to publication of the phylogenetic tree. The time taken for this was reduced during the period under consideration. The second approach would apply a constant, optimised time between sample date and tree publication. It would use the date of the most recent sample in each tree, after adjusting for the 99·9% quantile as described earlier, plus 8 days for sequencing and a further 2 days for the phylogenetics pipeline, instead of the tree publication date.

The TFP Scanner outputs a data frame with each row containing information on a particular cluster. We applied a variety of filters to these outputs as we selected subsets of phylogenomic clusters for analysis:

- Only extant clusters were retained. These were defined as clusters which had their most recent genomic sequence within 14 days of the most recent tip amongst the unfiltered set of clusters. This ensured that only contemporaneously circulating variants would contribute to the analysis.
- Non-external clusters were removed - an external cluster includes all the descendants of the cluster's most recent common ancestor (MRCA). However, we wanted to investigate whether incorporating larger clusters in our analysis would improve the performance of leading indicators. Therefore, in addition to having a set of leading indicators derived from a subset of clusters where the non-external clusters had been removed, we also created subsets where external clusters had been replaced by parent (non-external) clusters when the LGR of the parent cluster was at least ∈ { 65%, 70%, 75%, 80%, 85%, 90%, 95%, 100% } of that of the maximum LGR amongst the sub-clusters. The rationale being that the size of the cluster included in the analysis would be increased but the LGR, and therefore phenotypic characteristics, were comparable.
- A variable filter was applied to the LGR p-value. Three different filter thresholds were set ∈ {0·01, 0·05, 10,000 (i.e. no threshold)}.
- Clusters with overlapping tips were removed so as to maintain independence of cluster growth rates in our analysis.

This resulted in 720 unique sets of parameters for the set of phylogenetic trees that we investigated. In addition, we used each of these outputs to produce 19 different phylogeny-derived leading indicator time series (*summarised in Table S2*) and we assessed these against 101 different early warning signal threshold levels (0·00 to 5·00 in increments of 0·05). This gave us a total of 1·38 million EWS time series to evaluate.

TFP Scanner input parameter	Description	Constant or varied	Value(s)
Tre	ML phylogenetic tree	Varied	Set of 288 trees between 14 August 2020 and 29 March 2022
Amd	Tree metadata	Constant	
min_descendants	Clade must have at least this many tips	Varied	{ 20, 50, 100, percentage of samples between min_date and max_date* }
max_descendants	Clade can have at most this many tips	Constant	20e3
min_cluster_age_yrs	Only include clades that have sample tips that span at least this value	Varied	{ 7/365, 14/365, 28/365 }
min_date	Only include samples after this data	Varied	max_date - { 56, 84 }
max_date	Only include samples before and including this date	Varied	Date of most recent sample in tree after filtering for UK Pillar 2 samples and removing samples with erroneous and possibly erroneous dates
min_blen	Only compute statistics for nodes descended from branches of at least this length	Constant	1/30e3/2 (= 1.67e-5)
Ncpu	number cpu for multicore ops	Constant	1
num_ancestor_comparison	When finding comparison sample for molecular clock stat, make sure sister clade has at least this many tips	Constant	500
factor_geo_comparison	When finding comparison sample based on geography, make sure sample has this factor times the number within clade of interest	Constant	5
Tg	Approximate generation time in years. Growth rate is reported in these units.	Constant	6·5/365
report_freq	Print progress for every n'th node	Constant	50
mutation_cluster_frequency_threshold	If mutation is detected with more than this frequency within a cluster it may be called as a defining mutation	Constant	0·75
test_cluster_odds	A character vector of variable names in \code{amd}. The odds of a sample belonging to each cluster given this variable will be estimated using conditional logistic regression and adjusting for time.	Constant	c()
test_cluster_odds_value	Vector of same length as \code{test_cluster_odds}. This variable will be dichotomised by testing for equality of the variable with this value (e.g. vaccine_breakthrough == 'yes'). If NULL, the variable is assumed to be continuous (e.g. patient_age).	Constant	c()
root_on_tip	If input tree is not rooted, will root on this tip	Constant	'Wuhan/WH04/2020'
root_on_tip_sample_time	Numeric time that tip was sampled	Constant	2020
detailed_output	If TRUE will provide detailed figures for each cluster	Constant	FALSE
compute_gam		Constant	TRUE
compute_cluster_muts		Constant	FALSE

Table S1: Input parameters for TFP Scanner R package.
The majority of parameters were kept constant but some were varied in order to search for the best leading indicators derived from the TFP Scanner outputs. The parameters that were varied were the minimum number of descendants in clades (min_descendants), the time period from which to include samples (min_date, max_date), and the minimum age of the clade (min_cluster_age_yrs). * The percentage was calculated so as to set the minimum number of descendants across the time series to 20. This was equivalent to 0·3222688% of the minimum number of samples for the 56 day period and 0·2373887% for the 84 day period.

Phylogeny-derived leading indicators investigated

We derived 19 potential leading indicators (*summarised in Table S2*) from each of our 720 unique outputs from the TFP Scanner. Each potential leading indicator was derived using a relatively simple statistical analysis of either the cluster growth rate statistics or the evolutionary rate analysis computed by the TFP Scanner.

For each of the three cluster growth rate statistics (LGR, GAM LGR and simple LGR) we computed leading indicator time series separately using the maximum growth rate amongst clusters, the simple mean, the weighted mean (by cluster size) and the variance (weighted by cluster size) on both a sample and population basis.

The reason for investigating the maximum and mean cluster growth rates as a leading indicator is that higher growth rates are likely to lead to higher numbers of infections and therefore increased potential for an epidemic wave.

The rationale for investigating the variance of the cluster growth rates as a potential leading indicator is based on Fisher's fundamental theorem of natural selection, which states that "the rate of increase in fitness of any organism at any time is equal to its genetic variance in fitness at that time."[9] Applying Fisher's theorem to the SARS-CoV-2 pandemic, our suggestion is that the rate of increase in fitness of the virus may be represented by the rate of change in transmissibility. The latter is generally represented by the rate of change in the reproductive ratio (dR_t/dt) but for which we also use the second time derivative of UK hospitalisations ($d^2(new\ hospital\ admissions)/dt^2$) as a directly measurable proxy, albeit with additional time dependent confounding factors such as inherent virus severity, host immunity, admission of patients with or due to SARS-CoV-2 infection. In our study we represent the genetic variance in fitness of the virus by the variance of LGRs among clades within a large phylogeny reconstructed from SARS-CoV-2 sequences. Effectively our suggestion is that the growth rate can be considered a phenotype of the virus on the basis that it is a behavioural property influenced by genetic variation in the virus and the environment in which it finds itself. Our hypothesis was that higher levels of variation in LGRs are the result of increased genetic variation and that, according to Fisher's theorem, this implies a higher rate of increase in fitness of the virus, which should lead to increased transmission. While we saw some indication of positive correlation between the variance in cluster growth rates and the rate of change in the effective reproduction rate, we suspect that the strength of the correlation is impacted by confounding factors. As a result, the leading indicators based on the variance of cluster growth rates did not perform as well as other phylogeny-derived leading indicators in generating EWS for increases in hospitalisations.

An additional leading indicator using the variance of the cluster LGR was investigated, but this time dividing by the mean cluster size as a way of adjusting for the testing and sequencing capacity which varied significantly during the period under investigation. There is an issue here that when the number of samples is smaller the variance would be expected to be higher and so this is a confounding factor for this as a leading indicator. It is often the case that infections are low prior to a new wave of infections and so the number of sequences will be low and the variance higher for this reason rather than due to a genuine increase in the genetic variance. However, this can be investigated by comparison with situations when infections remain high but a new epidemic wave is caused by a new variant. Such a situation occurred when Omicron replaced Delta as the dominant variant in the UK. The number of infections due to the Delta variant oscillated at a relatively high level for a prolonged period in the second half of 2021 as NPIs had the effect of reducing the peak number of infections but extending the duration of the wave and producing several smaller peaks. While the number of Delta infections still remained high, it was supplanted as the dominant circulating variant by Omicron BA.1 in December 2021. Therefore elevated levels of variance in cluster LGRs in the period between September and December 2021 are less likely to be driven by lower sampling. The effect of sampling rate on leading indicators and early warning signals is also of broader interest from the point of view of future surveillance strategy, now that testing and sequencing capacity has been scaled down.

We also looked at time series for the maximum and mean of the absolute values for the MCO statistic. Higher than expected evolutionary rates indicate a potential increase in the fitness of the virus, which may lead to increased transmission and therefore increase the potential for an epidemic wave.

The final potential leading indicator was derived by identifying the Pango lineage[10] that was dominant (highest sample frequency within a cluster) in the most clusters and computing the maximum LGR among the clusters where this Pango lineage was dominant.

Leading indicator number	TFP Scanner output	Statistical measure
1	Logistic growth rate	Maximum
2		Simple mean
3		Weighted mean (by cluster size)
4		Sample variance
5		Population variance
6		Sample variance / mean cluster size
7	GAM logistic growth rate	Maximum
8		Simple mean
9		Weighted mean (by cluster size)
10		Sample variance
11		Population variance
12	Simple logistic growth rate	Maximum
13		Simple mean
14		Weighted mean (by cluster size)
15		Sample variance
16		Population variance
17	Molecular clock outlier (MCO)	Maximum of absolute values
18		Mean of absolute values
19	Dominant Pango lineage	Maximum logistic growth rate

Table S2: Summary of potential leading indicators derived from TFP Scanner outputs and ultimately from the SARS-CoV-2 pathogen via the phylogenetic tree expansion with time.

Calculation of early warning signals (EWS)

Within the literature, usage of the term 'Early Warning Signals (EWS)' varies. It can be generic or specifically refer to one or more of a commonly used set of statistical measures (e.g. variance, skewness, autocorrelation, and coefficient of variance, over a defined time period) most often reported on incidence or prevalence data, which we term 'direct data'. The use of these statistical measures is supported by the theory of 'critical slowing down',[11,12] which predicts that these statistical measures will undergo significant changes at the time of a critical transition. This theory has been applied within a range of different fields of study but in relation to infectious disease, significant changes in the summary statistics are expected to precede a rapid increase in cases at the beginning of a disease outbreak. In such circumstances the critical transition could be the change in the effective reproduction number: $R_{t_1} < 1 \rightarrow R_{t_2} > 1$. As we have primarily investigated 'indirect' data, for which it is not clear whether there would be such a critical transition, as a leading indicator we have not relied on this theory and so have used a simpler statistical measure for the change in our standardised leading indicators. Therefore, within this study we use the term early warning signals (EWS) in its generic sense; simply, a signal that represents an early warning.

We standardised the potential leading indicator time series described in the previous section by computing the 'robust' z-score, rather than the conventional z-score. The 'robust' z-score is calculated using the median rather than the mean (*see Equations S1 & S2*) and so is less sensitive to outliers.

$$'Robust'Z\ Score = \frac{(X_i - median(X))}{MAD}, \quad [S1]$$

where

$$MAD = Median\ Absolute\ Deviation = median(|X_i - median(X)|) \quad [S2]$$

We chose to use this method because some of the LGR variance leading indicator time series contain very high outlying values. This has a significant impact on the running mean in such cases, which results in a negligible value for the standard z-score for dates following an outlier and therefore introduces the potential to miss EWSs for time periods after outlying data points.

The standardised 'robust' z-score time series for each leading indicator was compared against a range of threshold levels (0·00 to 5·00 in increments of 0·05) in order to calibrate our method and also to investigate any link between the number of false positive EWS and the earliest true positive EWS. Measurement against the EWS threshold levels was made on an 'add-one-in' basis to simulate the real-time analysis that would take place if the method were being used as part of a surveillance programme. If the 'robust' z-score was above the threshold level for two consecutive data points we recorded an EWS. The requirement for more than a single consecutive data point above the threshold is to reduce the number of false positives.[13] Future studies could vary this requirement to investigate its impact.

Each EWS generated was assigned to a particular epidemic wave. The bins for each wave were defined as being between the date at which the previous wave peak of new hospitalisations had halved and the same time point for the wave of interest. Our rationale being that peaks in leading indicators would be finished by this time and it is likely that the next leading indicator peak is yet to develop.

In order to quantify the ability of a leading indicator to generate EWS, and to be able to compare leading indicators, it is necessary to define the start of a wave of SARS-CoV-2 infections.

We used the number of new coronavirus disease 2019 (COVID-19) hospital admissions in the UK rather than the number of new cases for defining waves of infection. The measurement of hospitalisations has been more consistently measured, during the pandemic period under investigation (August 2020 to March 2022), than the measurement of the number of new positive cases, which varied due to testing supply and demand in this period.[14] Therefore, new hospitalisations can be argued as being a more stable measure of the burden of infection in the UK population and is ultimately the metric against which surveillance strategies are seeking to provide an early warning. That being said, the number of hospitalisations will not only have been dependent on the total number of infections but also on the severity of the prevailing SARS-CoV-2 variant, and the level of immunity from vaccination and/or prior infection. In addition, it will include hospitalisation of patients *with* COVID-19 rather than *due* to it and so there will be some sampling bias as the hospital population is not representative of the general population. There is also a longer time lag between infection and hospitalisation, albeit an unknown and varying proportion of hospitalisations will be *with* COVID-19 rather than *due* to it.

We determined the start of a wave of hospitalisations as the date of the inflection point between two wave peaks (*see Table S3*). This is the earliest possible time for detection of a new wave of hospitalisations and so is a particularly stringent target for an EWS and therefore should be considered as more of a benchmark for comparison than a target. The method for defining the wave start dates also uses the full time series of hospitalisations over this period and so the start dates identified are earlier than would be the case if determined on an 'add-one-in' (real-time) basis. We calculated the inflection points in the waves of UK COVID-19 hospitalisations from the complete data set using an optimised generalised additive model (GAM)[13,15]. The GAM is a smoothed function representing the time series of the number of hospitalisations in the UK.

The daily time series of new COVID-19 hospitalisations and new cases in the UK between 23 March 2020 and 3 May 2022 was obtained from the UK's Coronavirus dashboard (https://coronavirus.data.gov.uk/) on 9 May 2022.[16] A GAM was fitted to the hospitalisation time series, using the *mgcv* package[17] in the R programming language, to facilitate a formal method of extracting a date that we could use as our definition of the beginning of a wave of SARS-CoV-2 infections.

A number of the parameters used in the GAM were varied in order to optimise the model by maximising the 'quality' of the fit, as measured using R^2, *k-index* and effective degrees of freedom *(edf) / k* ratio,[18] and the ability of the model to identify start dates for the major waves of hospitalisations during this period (B.1.177,

Alpha, Delta, Omicron BA.1, Omicron BA.2). The selected GAMs also identify three wave start dates within the broad wave of infections caused by the Delta variant, which had mini peaks and troughs.

The wave start dates were defined as the date at which the growth of the smoothed GAM of hospitalisations moves from negative to positive. Parameters that were varied during the GAM optimisation included the smoothing functions and the basis dimension, *k*, which sets the upper limit on the degrees of freedom. A number of criteria were set for selecting GAMs. Quality criteria included: *edf / k* ratio ≤ 0·9 and *k-index* ≥ 0·9. The other criterion was that the GAMs must identify start dates for five waves, after a low resolution filter had been applied. This was applied in order to avoid the requirement to identify start dates for the two smaller Delta wave peaks that followed its initial onset. The low resolution filter consisted of a wave peak-to-trough ratio cut-off level of 1·379, with the inverse (1/1·379 = 0·725) used for the trough-to-peak ratio cut-off level, and a peak-to-peak time cut-off of 55 days.

Predominant SARS-CoV-2 variant during wave of UK hospitalisations	Earliest date identified using generalised additive models (GAMs)	Latest date identified using generalised additive models (GAMs)	Range (days)	Wave start date used in comparison with EWS dates	
				**Based on UK new hospital admissions**	**Based on estimated value of R_t increasing above 1***
B.1.177	19 Aug 2020	-	-	19 Aug 2020	6 Sep 2020
Alpha	29 Nov 2020	-	-	29 Nov 2020	13 Dec 2020
Delta (1st wave)	10 May 2021	12 May 2021	3	11 May 2021	22 May 2021
Delta (2nd wave)	3 Aug 2021	-	-	3 Aug 2021	19 August 2021
Delta (3rd wave)	27 Sep 2021	28 Sep 2021	2	27 Sep 2021	13 Oct 2021
Omicron BA.1	25 Nov 2021	27 Nov 2021	3	26 Nov 2021	25 Nov 2021
Omicron BA.2	21 Feb 2022	22 Feb 2022	2	21 Feb 2022	11 Mar 2022

Table S3: **COVID-19 epidemic wave start (inflection) dates in the UK.** Dates computed using (a) new hospital admissions, and (b) estimated values of the time varying, or effective, reproduction number, or reproductive ratio, R_t to determine the date of 'critical transition' when R_t increases above a value of 1. *Using the midpoint in the confidence interval, with bi-weekly data transformed to daily data using a generalised additive model (GAM) and spline interpolation.

A total of 2,860 GAMs were generated with 12 meeting these criteria, all of which had R^2 values in excess of 0·994. The smoothing functions used in these 12 GAMs were thin plate (tp), thin plate with modified smoothing penalty (ts), Duchon spline (ds), P-spline (ps), Gaussian process (gp), cubic regression spline (cr) and shrinkage cubic regression spline (cs). The basis dimension (*k*) ranged from 130 to 170. All 12 GAMs generated the same wave start dates for the B.1.177 and Alpha waves, while start dates for Omicron BA.2 were within one day and two days for Omicron BA.1 and the first Delta wave. In relation to the change in fitness of the virus, the wave start dates for the smaller Delta peaks after the initial onset are of secondary importance as they are a continuation of the first Delta peak. Nevertheless the start dates identified by the 12 GAMs for the first of these two smaller Delta waves were in agreement and the dates for the second were within two days.

Hospitalisation numbers are also impacted by the day of the week that they are reported. This was taken into account using a weekday element in the GAM with a cyclic cubic spline with basis dimension $k = 7$.

We also calculated the lead (or lag) times for the EWS relative to the critical transition[19] of the effective reproduction number, when $R_{t_1} < 1 \rightarrow R_{t_2} > 1$ (*see Table S4*). However, we would note that this is an estimated value rather than a direct measure. We sourced the time varying, or effective, reproduction number, or reproductive ratio, R_t dataset from the UK Coronavirus dashboard (https://coronavirus.data.gov.uk/)[16] on 20 December 2022. We transformed this weekly dataset, which provides a likely range for the effective reproduction number, by taking the midpoint of the range and using a GAM, with thin plate spline and basis dimension $k = 100$, to smooth the data set and a spline to interpolate and expand to a daily time series.

An important part of evaluating methods for generating EWS is differentiating between true and false positive signals. One way to do this is to define a time window around the event, such as a critical transition, and classify EWS within this window as a True Positive.[19] We set a time window around each epidemic wave to determine which wave the EWS belonged to. However, the sequence data that we used to generate the EWS enables us to be more specific in our true or false positive classification method. We classified EWS within these time windows as being a true or false positive based on whether the EWS was driven by the same SARS-CoV-2 variant, as determined by the designated Pango lineage[10] including sub-lineages, that drove infections. A true positive was recorded where a match was made and a false positive where there was no match.

We identified the SARS-CoV-2 variant representing the largest proportion of sequences in each phylogenetic cluster identified by the TFP Scanner for each tree, and with each set of scan parameters as described earlier. These variants were then ranked by the number of clusters in which they were the dominant variant (the "dominant variant ranking"). We also ranked the clusters by their LGRs (the "growth rate ranking"), including GAM and simple LGRs as described earlier. Only positive growth rates were included in the ranking as we are only interested in variants that were growing at the time an EWS was generated. If the predominant variant in the UK at the time, in terms of infections, appeared in the top 5 of either the dominant variant ranking or the growth rate ranking then we classified this as a true positive EWS. All other EWS were classified as false positives.

Assessment of leading indicators

The first criteria in our assessment is that a parameter set must generate at least one true positive (TP) EWS for each of the seven epidemic waves during the period investigated. This filter reduced the number of parameter sets to 40,720.

We calculated the lead times between the earliest TP EWS and the epidemic wave start (inflection point) dates. We also recorded the number of false positive (FP) EWS and split them between those before and those after the earliest TP EWS. Our rationale being that it is more important to minimise the number of FP EWS before the first TP EWS.

We used these two metrics in a variety of ways to rank the leading indicators and parameter sets. In terms of lead time, we used two separate criteria: (1) total lead time across all waves; (2) total lead time across waves driven by genomic variation (Alpha, Delta (first peak), Omicron BA.1 and Omicron BA.2). We did not include the first epidemic wave in the period investigated, when B.1.177 was dominant, in this latter set of waves as we have limited data prior to our calculated wave start date; the first phylogenetic tree that we have is dated 14 August 2020 and the B.1.177 wave start date is 19 August 2020. Therefore, it is possible that had our leading indicator time series extended further back in time, earlier TP EWS would have been generated for this wave.

Our understanding of current SARS-CoV-2 surveillance strategy is that maximising lead time is of greater importance than minimising FPs. This is because any EWS generated would be used as a prompt for further in-depth analysis prior to any policy change. However, we also investigated filtering out parameter sets based on the number of false positives before ranking by lead time. In addition to not limiting the number of FP EWS we filtered for a maximum of 0, 2, 5, and 10 FP EWS, for an individual wave. We also split this between the number of FPs in total and the number of FPs prior to the earliest TP EWS. Another filter was applied that required the earliest TP EWS for each wave to either be before the wave start (inflection) date or be equal to the earliest TP EWS amongst the set with a TP EWS for all seven waves. These limits were also applied separately to the number of FPs before the earliest TP per wave.

			B.1.177	Alpha	Delta (1)	Delta (2)	Delta (3)	Omicron BA.1	Omicron BA.2
**Epidemic (hospitalisations) wave start (inflection) date**			19 Aug 2020	29 Nov 2020	11 May 2021	3 Aug 2021	27 Sep 2021	26 Nov 2021	21 Feb 2022
R_t critical transition date			6 Sep 2020	13 Dec 2020	22 May 2021	19 Aug 2021	13 Oct 2021	25 Nov 2021	11 Mar 2022
Earliest True Positive EWS date			26 Aug 2020	23 Nov 2020	21 Apr 2021	4 Aug 2021	18 Sep 2021	2 Dec 2021	4 Feb 2022
EWS lead time (days) relative to: Lead (-ve) and Lag (+ve)	Hospitalisation wave start (inflection) date		+7	-6	-20	+1	-9	+6	-17
	R_t critical transition date		-11	-20	-31	-15	-25	+7	-35
Number of False Positives	Prior to earliest True Positive		0	0	2	0	0	2	0
	After earliest True Positive		4	0	0	0	0	0	0
Positive Predictive Value i.e. Precision			0·76	1·00	0·95	1·00	1·00	0·90	1·00
Change in number of daily hospital admissions	between EWS and wave start (inflection) date	Number	-17	-200	-64	-17	+23	-106	-178
		%	-13%	-13%	-37%	-9%	+3%	-13%	-13%
	between EWS and wave peak	Number	+1843	+2990	+2432	+1843	+406	+1795	+1263
		%	+1418%	+188%	+1406%	+40%	+53%	+214%	+89%

Table S4: Early warning signals (EWS) generated by selected phylogeny-derived leading indicators for COVID-19 waves of infection in the UK. Data shown is the same as in Table 1 in the main article, with the addition of lead and lag times relative to the R_t critical transition. The earliest lead time was 35 days and the longest lag time was 7 days. The mean lead time across the seven waves was 18·6 days.

After the application of the various filters, the remaining parameter sets were ranked on the basis of lead times across all seven waves and, separately, across the four genomic variant driven waves with sufficient data prior to the wave start (Alpha, 1st Delta, Omicron BA.1 and BA.2 waves). A summary of the different filters and ranking criteria is shown in Table S5.

Table 3 in the main article shows the best leading indicator and parameter set that we selected for each set of filters and ranking criteria. The selection of a single best performing parameter set requires some subjective analysis and judgement as more than one parameter set will often achieve the same ranking score under the various criteria. Broadly speaking, in making our subjective choices we considered the overall performance of the parameter sets in terms of lead time and number of FP EWS. For example, two parameter sets may have the same total lead time across the seven epidemic waves, but the lead times may vary across the individual waves. An extreme hypothetical example of this could be that one parameter set generates EWS with a lead time of 20 days for wave A and a lag time of 20 days for Wave B, which would achieve the same ranking score for total lead time as a parameter set generating a lead time of 0 days across both waves. In such a situation, we would favour the latter parameter set as it generates a useful EWS for both waves, but we recognise that different observers may select different parameter sets as being the 'best' performers under each set of ranking criteria. The top results for each set of filter and ranking criteria are therefore shown in a file (SM1–Best EWS Results by Filter and Ranking Criteria.xlsx) as part of the Supplementary Materials.

We also calculated the precision (*see equation S3*), also known as the positive predictive value (PPV), of each parameter set for each wave and as an average per wave. While we did not use this as a formal filter or ranking criteria we did consider it where a subjective choice was made.

$$Precision = \frac{Number\ of\ True\ Positive\ EWS}{(Number\ of\ True\ Positive\ EWS + Number\ of\ False\ Positive\ EWS)} = \frac{TP}{(TP+FP)} \qquad [S3]$$

**Filter or ranking criteria**	**Set of variables applied**
**Filters**	
Number of true positive EWS for each wave	> 0
Number of false positive EWS for each wave	= 0, 2, 5, 10, any
Which false positive EWS to apply limits to	All, or only those before the earliest true positive EWS
Restrictions on individual wave true positive EWS lead time	All, or EWS before wave start date and this is not fulfilled the EWS date must be equal to the earliest EWS for that wave amongst parameter sets producing at least one true positive EWS for all seven waves.
**Ranking criteria**	
Lead time	All, or those driven by new genomic variant (Alpha, 1st Delta, Omicron BA.1 & BA.2)
Waves to include	All, or those driven by new genomic variant (Alpha, 1st Delta, Omicron BA.1 & BA.2)

Table S5: Filters and ranking criteria applied to EWS results from parameter sets.

Comparison between phylogeny- and non-phylogeny-derived leading indicators

During the COVID-19 pandemic, a variety of other datasets have been suggested as providing useful inputs into models for monitoring the dynamics of the epidemic waves. In this study we applied broadly the same methodology, used to generate EWS from our phylogeny-derived leading indicators, to a variety of potential leading indicators derived from non-phylogeny datasets. We compared lead times for EWS generated from all of these potential leading indicators. It should be noted that the EWS generation methodology was developed with a focus on the phylogeny-derived leading indicators and was then applied to the non-phylogeny-derived

leading indicators with some adjustments to make comparison possible. Therefore, better EWS lead times may be obtainable from the non-phylogeny-derived leading indicators if more focus were to be placed on them in the development of the methodology.

The non-phylogeny-derived leading indicators we investigated were various polymerase chain reaction (PCR) cycle threshold (Ct)[20-24] derived values, COVID-19 Pillar 2 test positivity rate, behavioural changes (Google mobility at various settings,[25] CoMix survey age-stratified mean contacts[26]) and time-shifted hospital admissions data[16] as a control test for the methodology. All of these data sets relate to the UK, apart from the COVID-19 test positivity rate and PCR Ct values which only relate to England.

In calculating the COVID-19 test positivity rate, we only included Pillar 2 community testing. Both lateral flow tests (LFT) and polymerase chain reaction (PCR) tests were included in the calculation. The test positivity rate was calculated as the number of positive tests recorded each day divided by the sum of positive and negative tests. While the expectation would be that the positivity rate increases as the number of infections increase within the population, the positivity rate is also affected by changes in the overall number of tests. It could therefore be impacted by changes in behaviour towards symptomatic and asymptomatic testing.[27]

Ct values determined during PCR tests can be used as a proxy for SARS-CoV-2 viral load.[28] PCR Ct values are inversely proportional to the viral load during an infection with every ~3.3 change in Ct value reflecting 10-fold difference in the amount of virus.[29] The expectation is that the median Ct value is lower (higher viral load) amongst tests earlier in an epidemic as more people have new infections (viral load drops during an infection) and the skewness of the distribution also changes during growth and fall of an epidemic.[20] We used data collected from samples in England under Pillar 2 community testing. This avoided the bias seen in Pillar 1 hospital testing towards lower Ct values (higher viral load) as might be expected for patients hospitalised as a result of infection. We derived 36 potential leading indicators from the Ct value data, including: the raw Ct values for three different gene targets (N, ORF1ab and S); normalising for the reference (or control) Ct value in each sample (samples with no reference Ct value were excluded from the analysis); converting to an estimated viral load value using equation S4.[28] We also took the mean, minimum and maximum values for the three gene targets, as test results for some variants were impacted by target gene dropout. This was first seen in the S-gene target failure (SGTF) for the Alpha variant. In addition to the impact of target gene dropout, the level of Ct values can also vary by virus variant.[30] To try to eliminate this confounder across multiple waves and variants, we derived additional leading indicators by calculating the skewness and standard deviation of the distribution of values for each day. The full set of Ct value derived potential leading indicators are shown in Table S6.

$$Viral\ load = ln(2^{-\Delta C_t}) = ln\left(2^{-\left(C_{t_{target}} - C_{t_{reference}}\right)}\right) \quad [S4]$$

Behavioural data can potentially be a leading indicator as more mixing within the population is likely to lead to increased transmission of infection. We investigated behavioural data as a leading indicator for epidemic waves using two datasets: Google mobility and the CoMix behavioural survey. Google mobility[25] measures the number of people visiting (or the time spent) in various different categories of place. The data made available is the percentage change relative to a baseline period, which is the median daily value between 3 January and 6 February 2020. We took a 7-day rolling mean of this data to smooth for changes due to the day of the week, but we did not make any adjustments for seasonality or public holidays, both of which are likely to have a material impact on the values. The CoMix behavioural survey data[26] measures the mean number of daily contacts on a weekly basis. The data are stratified across a range of demographic factors, but we only used the age group stratification to derive potential leading indicators.

We also included the UK hospital admissions data[16] with time shifts applied as a control test for the analysis method used for the phylogeny-derived leading indicator data. This dataset is less relevant here, particularly when leading indicators are compared against the R_t critical transition rather than the hospitalisation wave start (inflection point) dates.

These data sets have data points over different time ranges and different sampling rates, with some daily and others weekly or bi-weekly. To make them comparable we filled in data for missing dates using a simple linear interpolation. We then trimmed all of the data sets to the maximum range for which we had data across all potential leading indicators. This approach was also applied to the phylogeny-derived leading indicators for comparison with the non-phylogeny-derived leading indicators, but not when the former were considered alone.

Dataset	Description	Potential leading indicators investigated	
PCR cycle threshold (Ct) levels	The cycle threshold (Ct) value determined during a polymerase chain reaction (PCR) test can be used as a proxy for viral load [ref]. Ct values were used from pillar 2 community testing in the UK. Lower Ct values are associated with higher viral loads.<br>Ct values for three different gene targets were used. Separately, we normalised these Ct values for the control value (normalised gene Ct value = gene specific Ct value - control Ct value). We also calculated the viral load proxy for each gene target as a potential leading indicator. In addition, we calculated summary statistics for use as leading indicators. This was repeated for the mean value across samples for each day as well as the median value.	Ct value for ORF1ab gene (mean and median of daily values)<br>Ct value for N gene (mean and median of daily values)<br>Ct value for S gene (mean and median of daily values)<br>Ct value for control (mean and median of daily values)<br>Minimum Ct value for O, N, S genes in each sample (mean and median of daily values)<br>Mean of Ct values for O, N, S genes in each sample (mean and median of daily values)<br>Normalised Ct value for O gene (mean and median of daily values)<br>Normalised Ct value for N gene (mean and median of daily values)<br>Normalised Ct value for S gene (mean and median of daily values)<br>Viral load calculated from O gene Ct value (mean and median of daily values)<br>Viral load calculated from N gene Ct value (mean and median of daily values)<br>Viral load calculated from S gene Ct value (mean and median of daily values)<br>Minimum viral load value for O, N, S genes in each sample (mean and median of daily values)<br>Mean viral load value for O, N, S genes in each sample (mean and median of daily values)<br>Maximum viral load value for O, N, S genes in each sample (mean and median of daily values)<br>Skewness of the daily distribution of the minimum Ct value for O, N, S genes in each sample<br>Standard deviation of the daily distribution of the minimum Ct value for O, N, S genes in each sample<br>Skewness of the daily distribution of the maximum viral load values for O, N, S genes in each sample<br>Standard deviation of the daily distribution of the maximum viral load values for O, N, S genes in each sample	
Google mobility	Percentage change in the number of visitors to (or time spent in) categorised places compared to the baseline, which is the five-week period from 3 January to 6 February 2020.<br>Data for six different categories of place were available. We added a seventh which we calculated as the inverse of the Parks category data. The percentage change as well as the 7-day rolling mean of the percentage change were investigated in the analysis.	Grocery & pharmacy<br>Parks<br>Parks (inverse)<br>Residential<br>Retail and recreation<br>Transit stations<br>Workplaces	Grocery 7-day rolling mean<br>Parks 7-day rolling mean<br>Parks (inverse) 7-day rolling mean<br>Residential 7-day rolling mean<br>Retail and recreation 7-day rolling mean<br>Transit stations 7-day rolling mean<br>Workplaces 7-day rolling mean
CoMix survey	Mean number of daily contacts as measured by a weekly survey in the UK. Data includes stratification by age group.	All age groups<br>All adults<br>Ages 0-4 years<br>Ages 5-11 years<br>Ages 5-17 years<br>Ages 18-29 years<br>Ages 18-59 years	Ages 30-39 years<br>Ages 40-49 years<br>Ages 50-59 years<br>Ages 60-69 years<br>Ages 60 years and over<br>Ages 70 years and over
New hospital admissions	Daily new hospital admissions in the UK with a time shift applied to use as a control to check the method used in the genomic leading indicator analysis, but also included here.	0-day time-shift<br>10-day time-shift	20-day time-shift<br>30-day time-shift
COVID-19 test positivity rate	Pillar 2 community testing in England (lateral flow and PCR test)	Positivity rate	7-day rolling mean of positivity rate

Table S6: Non-phylogeny-derived leading indicators investigated.

All of these potential leading indicator datasets have a time lag between the date of the last data point, or the time taken for data to be included in the set, and the publication date. To try to adjust for this in our attempt to simulate real time EWS generation, we added 7 days to the hospitalisation data, the Ct value data and the test positivity rate data. We added 3 days to the Google Mobility data, which is stated to be published 2-3 days after the fact. For the CoMix data we added 10 days; comprising 7 days due to the bi-weekly basis of the dataset, which uses the mid-point of the two-week period, and a further 3 days for analysis and publication.

Clearly, the classification method for True or False Positives described earlier involving matching the variant contributing to the EWS signal with the variant driving the epidemic waves is not possible for the potential leading indicators that were not derived from the SARS-CoV-2 phylogeny. We therefore used a time window for such leading indicators; a common approach often used for 'direct' data. We also applied this as a secondary method to the phylogeny-derived leading indicators so as to make them comparable to the non-phylogeny-derived leading indicators. We made some further adjustments to the phylogeny-derived leading indicator data sets to make them comparable. The time series were expanded to daily, with a simple linear interpolation used to fill values for missing dates. We also trimmed the time series date range to match that of the maximum range to which all phylogeny-derived and non-phylogeny-derived leading indicators had data.

We used time windows starting 30 days before our defined wave start (inflection) date, to limit overlap with previous waves, and ending 5 days after, to account for small possible bifurcation delays. This is in line with the time window used by Proverbio et al.[19] We also looked at results using a time window of $-30 < t < +10$ days, as we knew that for some waves the earliest true positive EWS for phylogeny-derived leading indicators was more than 5 days after the wave start (inflection) date. For this part of the analysis, we dropped the requirement for two consecutive data points to be above the threshold before recording an EWS. This was to enable the calculation of receiver operating characteristic (ROC) statistics with a more straightforward calculation and interpretation of True Positives (TP), False Positives (FP), True Negatives (TN), and False Negatives (FN). Therefore any data point above the EWS threshold and within the time window was recorded as a TP and any below the threshold within this time window were FNs. We applied the reverse classification outside the time window; FP above the threshold and TN below. We repeated this method using the R_t critical transition date as an anchor point for the time window. While this method enables comparison between the various potential leading indicators it is clear that some EWS will be classified in a different category compared with the method that tries to match the variant generating the signal and that driving the epidemic wave. One possible way to reconcile this is that True Positives (TPs) using this matching method that occur more than 5 days (or 10 days) after the wave start (inflection) date and/or R_t critical transition date could be considered to be too late to be a useful EWS and so may as well be considered to be a False Positive.

We also applied a different ranking process to compare the phylogeny-derived leading indicators with the non-phylogeny-derived leading indicators that we investigated. Using the classification of the 'robust' z-score values we were able to calculate a range of ROC statistics, including the True Positive Rate (*equation S5*), also known as the Sensitivity, and False Positive Rate (*equations S6 & S7*).

$$True\ Positive\ Rate = Sensitivity = \frac{TP}{TP + FN} = \frac{Number\ of\ True\ Positive}{Number\ of\ True\ Positive + Number\ of\ False\ Negative} \quad [S5]$$

$$False\ Positive\ Rate = \frac{FP}{FP + TN} = \frac{Number\ of\ False\ Positive}{Number\ of\ False\ Positive + Number\ of\ True\ Negative} \quad [S6]$$

$$= 1 - Specificity = 1 - \frac{TN}{FP + TN} \quad [S7]$$

Calculating these ratios across a range of EWS thresholds (-5·0 to +5·0 in increments of 0·05) we were able to plot a ROC curve and calculate the area under the curve (AUC). We calculated the ROC AUC for individual waves as well as for time periods covering multiple waves: all seven waves; a period excluding the B.1.177 wave; and a period excluding the B.1.177 wave and the Omicron BA.2 wave. We have limited data in our leading indicator time series ahead of the B.1.177 wave start date and limited data after the start of the Omicron BA.2 wave start date, for some leading indicators investigated.

We used a variety of measures to assess the ability of each leading indicator to generate EWS over the period. These included the ROC AUC as well as the Matthews Correlation Coefficient (MCC),[31] shown in equation S8,

which has been used in various fields and has been suggested as a more discriminative measure of binary classification assessment.[32] A high value for the MCC, which ranges from -1 to +1, can only be achieved with high values in all four basic rates of the confusion matrix (Sensitivity, Specificity, Precision and Negative Predictive Value), whereas a high value for the ROC AUC can be produced without values for Precision (aka Positive Predictive Value) or Negative Predictive Value necessarily being high. We also calculated the normalised version of the MCC (*see equation S9*), which ranges from 0 to +1 making it more comparable to the ROC AUC.

$$Matthews\ Correlation\ Coefficient\ (MCC) = \frac{(TP \cdot TN) - (FP \cdot FN)}{\sqrt{(TP+FP) \cdot (TP+FN) \cdot (TN+FP) \cdot (TN+FN)}} \quad [S8]$$

$$Normalised\ MCC = \frac{MCC + 1}{2} \quad [S9]$$

We also calculated the F1 and Fowlkes-Mallows Index which are more focused on positive results.

This method was applied to the 19 phylogeny-derived leading indicators shown in Table S2, with the same TFP Scanner parameter sets and filters described earlier, and the 71 non-phylogeny-derived leading indicators shown in Table S6. The number of EWS thresholds was increased from 101 (0·00 to +5·00 in increments of 0·05) to 201 (-5·00 to +5·00 in increments of 0·05) and we investigated four true positive time windows as described earlier. This resulted in a total of 11·1 million individual leading indicator parameter sets to be assessed.

In filtering and ranking these leading indicator parameter sets, we excluded performance in the B.1.177 and Omicron BA.2 waves, due to there not being sufficient data points before and after, respectively, the wave start (inflection) and/or R_t critical transition dates. Our performance assessment was therefore focused on the Alpha, 1st, 2nd and 3rd Delta, and Omicron BA.1 waves. Firstly, we filtered for leading indicator parameter sets that produced a normalised MCC value for each of these five waves individually. This reduced the number of leading indicator parameter sets to 899,296. Normalised MCC's were typically not defined due to there either being no positives or no negatives for one or more individual waves. In order to assess the consistency of EWS performance, we computed the minimum value and the arithmetic mean of the normalised MCC across three sets of individual waves: all five waves with sufficient data (Alpha, Delta (1,2,3), and Omicron BA.1); waves driven by new genomic variants (Alpha, Delta (1), and Omicron BA.1); and waves not driven by new genomic variants (Delta(2,3)). We then identified the best leading indicators for each of these sets of waves by ranking them using the mean percentile rank based on the minimum and arithmetic mean of the normalised MCC across the waves. The top 5,000 ranked leading parameter sets can be found in a separate file (SM2–TFPS_vs_nonTFPS_Perc_Rank_Mean_Min_MCC.xlsx) as part of the Supplementary Material.

Table S7 shows the top ranked leading indicators based on all waves with sufficient data: Alpha, Delta (1,2,3), and Omicron BA.1. The leading indicators derived from phylogenomic data and non-phylogenomic data judged to have the best EWS performance are plotted in Figure S1. In relation to the non-phylogeny-derived leading indicators, the Google mobility workplaces leading indicator ranked slightly higher than the Google mobility grocery & pharmacy leading indicator, however, we have shown the latter in Figure S1 because, in our view, the normalised MCC results for the former are impacted by the periodicity of the weekday/weekend values. This is reflected in the Google mobility workplaces 7-day mean leading indicator, which smooths this periodicity, with the same EWS threshold value having a much lower ranking (93rd percentile vs 99th percentile, with mean normalised MCCs of 0·59 and 0·64, and minimum normalised MCCs of 0·44 and 0·54 respectively).

While the highest ranked non-phylogeny-derived leading indicators are preceded by a large number of phylogeny-derived leading indicators, they are not far behind in terms of percentile rank. However, from Figure S1 and Table S7 it can be seen that the best phylogeny-derived leading indicator outperforms the best non-phylogeny-derived leading indicator using this method of generating EWS and assessing them. The normalised MCC and ROC AUC values are higher, and the proportion of TP and TN is visibly higher in Figure S1.d than in S1.e.

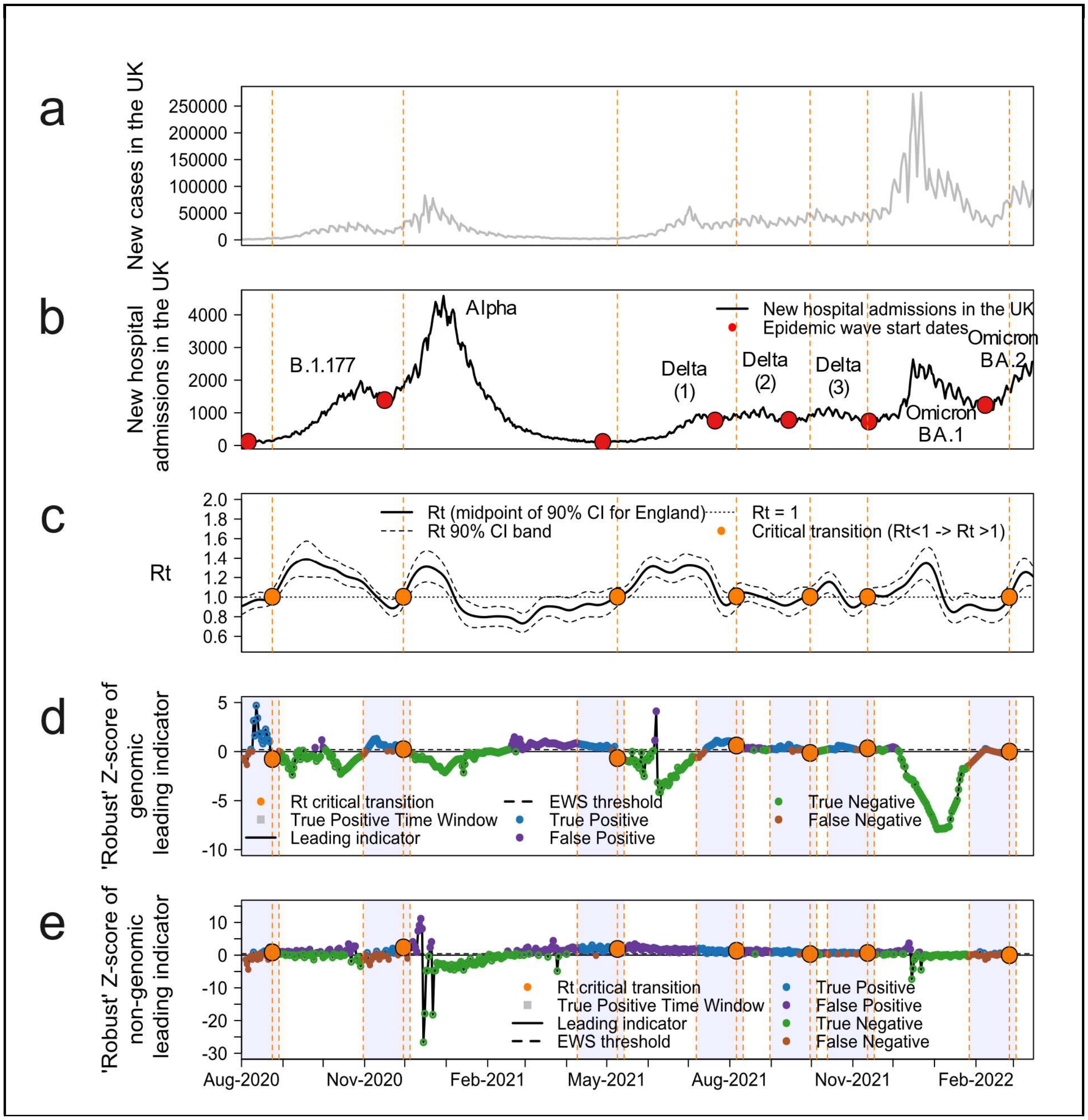

Figure S1: Early warning signals for COVID-19 epidemic waves in the UK assessed using 'time window' method. (a) New recorded positive cases of COVID-19 in the UK. (b) New COVID-19 hospital admissions in the UK with epidemic wave start (inflection) dates marked. (c) Estimated COVID-19 effective reproduction number (R_t) with 90% confidence intervals and 'critical transitions' (when R_t increases above 1) marked. (d) 'Robust' Z-score for one of the best performing leading indicators derived from the SARS-CoV-2 phylogeny (mean cluster logistic growth rate). EWSs are marked as true positive, false positive, true negative or false negative depending on whether they are above or below the EWS threshold and inside or outside the time window (in this case, -30 < R_t critical transition date < +5 days). (e) as in (d) but showing the best selected leading indicator derived from non-phylogenomic data (Google mobility grocery & pharmacy). Details of the leading indicator parameter values are shown in Table S7.

Analysis Pipeline

The analyses described here were made using the R programming language (version 4.2.0). The code and a summary of the pipeline is available here https://github.com/KieranODrake/Early_Warning_Signal.

Rank	Time window anchor	Leading indicator type	Leading indicator	Cluster min age	Cluster max age	Cluster min descendants	Parent/sub-cluster LGR replacement threshold (%)	P-value threshold	EWS threshold	B.1.177		Alpha		Delta (1)		Delta (2)		Delta (3)		Omicron BA.1		Omicron BA.2		Min norm. MCC Alpha to BA.1	Percentile rank	Arithmetic mean norm. MCC Alpha to BA.1	Percentile rank
										Norm. MCC	ROC AUC	Norm. MCC	ROC AUC	Norm. MCC	ROC AUC	Norm. MCC	ROC AUC	Norm. MCC	ROC AUC	Norm. MCC	ROC AUC	Norm. MCC	ROC AUC				
1	R_t+5	Genomic	Mean LGR	7	84	100	70	0.05	0.20	0.77	0.81	0.93	1.00	0.63	0.59	0.69	0.89	0.73	0.76	0.72	0.48	0.57	0.82	0.63	100.00	0.74	100.00
2	R_t+5	Genomic	Mean GAM LGR	7	84	100	65	0.05	0.25	0.75	0.82	0.81	1.00	0.63	0.60	0.81	0.95	0.65	0.67	0.79	0.54	0.55	0.91	0.63	100.00	0.74	100.00
3	R_t+5	Genomic	Mean LGR	14	84	100	60	0.05	0.15	0.77	0.82	0.91	1.00	0.62	0.60	0.73	0.88	0.63	0.76	0.81	0.49	0.63	0.73	0.62	100.00	0.74	99.99
4	R_t+5	Genomic	Mean LGR	14	84	100	70	0.05	0.20	0.76	0.82	0.88	1.00	0.61	0.59	0.80	0.93	0.72	0.76	0.74	0.50	0.57	0.82	0.61	99.99	0.75	99.99
5	R_t+5	Genomic	Mean GAM LGR	7	84	100	65	0.05	0.20	0.78	0.82	0.93	1.00	0.63	0.60	0.72	0.95	0.64	0.67	0.78	0.54	0.57	0.91	0.63	100.00	0.74	99.99
6	R_t+5	Genomic	Mean GAM LGR	7	84	100	60	0.05	0.20	0.74	0.82	0.88	1.00	0.64	0.61	0.69	0.91	0.63	0.68	0.83	0.57	0.59	0.82	0.63	100.00	0.73	99.99
7	R_t+5	Genomic	Mean GAM LGR	7	84	100	70	0.05	0.20	0.74	0.82	0.93	1.00	0.62	0.61	0.76	0.93	0.63	0.69	0.77	0.50	0.55	0.82	0.62	99.99	0.74	99.99
8	R_t+5	Genomic	Mean GAM LGR	7	84	100	70	0.05	0.20	0.79	0.84	0.80	1.00	0.61	0.63	0.96	0.98	0.73	0.80	0.65	0.45	NA	1.00	0.61	99.99	0.75	99.99
9	R_t+5	Genomic	Mean GAM LGR	7	84	100	75	0.05	0.25	0.75	0.80	0.84	0.99	0.61	0.62	0.78	0.93	0.67	0.71	0.77	0.51	NA	0.82	0.61	99.99	0.73	99.99
10	R_t+5	Genomic	Mean GAM LGR	14	84	100	80	0.05	0.25	0.73	0.81	0.80	0.99	0.61	0.61	0.86	0.96	0.71	0.72	0.73	0.49	NA	0.73	0.61	99.99	0.74	99.99
11	R_t+5	Genomic	Mean LGR	14	84	100	60	0.05	0.20	0.78	0.82	0.88	1.00	0.62	0.60	0.69	0.88	0.69	0.76	0.75	0.49	0.59	0.73	0.62	100.00	0.73	99.99
12	R_t+5	Genomic	Mean GAM LGR	7	84	100	70	0.05	0.30	0.77	0.82	0.78	1.00	0.63	0.61	0.85	0.93	0.67	0.69	0.70	0.50	NA	0.82	0.63	100.00	0.73	99.99
13	R_t+5	Genomic	Mean GAM LGR	14	84	100	70	0.05	0.25	0.75	0.83	0.80	1.00	0.61	0.61	0.86	0.96	0.66	0.69	0.79	0.56	NA	0.82	0.61	99.99	0.74	99.99
14	R_t+5	Genomic	Mean GAM LGR	7	84	100	60	0.01	0.25	0.74	0.78	0.78	1.00	0.63	0.60	0.81	0.91	0.65	0.63	0.77	0.61	0.62	1.00	0.63	100.00	0.73	99.99
15	R_t+5	Genomic	Mean GAM LGR	14	84	100	60	0.05	0.25	0.75	0.83	0.80	1.00	0.62	0.61	0.85	0.94	0.66	0.69	0.72	0.55	0.55	0.91	0.62	99.99	0.73	99.99
16	R_t+5	Genomic	Mean GAM LGR	14	84	100	65	0.05	0.25	0.75	0.83	0.78	1.00	0.61	0.61	0.81	0.94	0.67	0.68	0.79	0.57	NA	0.91	0.61	99.99	0.73	99.99
17	R_t+5	Genomic	Mean GAM LGR	14	84	100	80	0.05	0.20	0.74	0.81	0.81	0.99	0.61	0.61	0.80	0.96	0.69	0.72	0.74	0.49	0.55	0.73	0.61	99.99	0.73	99.99
18	R_t+5	Genomic	Mean GAM LGR	14	84	100	75	0.05	0.20	0.74	0.82	0.85	0.99	0.61	0.62	0.82	0.94	0.64	0.71	0.76	0.50	0.55	0.82	0.61	99.99	0.74	99.99
19	R_t+5	Genomic	Mean GAM LGR	7	84	100	80	0.05	0.20	0.74	0.80	0.87	0.99	0.62	0.61	0.73	0.96	0.68	0.71	0.74	0.45	NA	0.82	0.62	99.99	0.73	99.99
20	R_t+5	Genomic	Mean LGR	14	84	100	80	0.05	0.15	0.74	0.80	0.91	0.99	0.61	0.59	0.71	0.93	0.71	0.77	0.70	0.42	0.59	0.73	0.61	99.99	0.73	99.99
…	…	…	…	…	…	…	…	…	…	…	…	…	…	…	…	…	…	…	…	…	…	…	…	…	…	…	…
**Highest ranked non-phylogenetic-derived leading indicators**																											
3506	R_t+5	Non-genomic	Google mobility: workplaces	NA	NA	NA	NA	NA	0.30	0.52	0.48	0.66	0.82	0.67	0.67	0.54	0.36	0.59	0.60	0.76	0.78	0.42	0.44	0.54	99.60	0.64	99.22
3869	R_t+5	Non-genomic	Google mobility: grocery & pharmacy	NA	NA	NA	NA	NA	0.45	0.38	0.31	0.70	0.70	0.65	0.68	0.59	0.49	0.56	0.76	0.67	0.64	0.60	0.57	0.56	99.80	0.63	99.14
3909	R_t+10	Non-genomic	Google mobility: workplaces	NA	NA	NA	NA	NA	0.30	0.56	0.54	0.68	0.87	0.69	0.68	0.52	0.32	0.60	0.68	0.78	0.77	0.42	0.44	0.52	99.12	0.65	99.13

Table S7: Highest ranked genomic- and non-genomic-derived leading indicators. Ranking is by percentile for the arithmetic mean and minimum values for the normalised MCC across the Alpha, Delta (1,2,3) and Omicron BA.1 waves. The normalised MCC is calculated using the four elements of the confusion matrix (TP, FP, TN, FN) as defined using a time window anchored around either the wave start date or the R_t critical transition date and with a variable end date (+5 or +10 days). The data shown is an extract from the top 5,000 ranked leading indicator parameter sets (SM2–TFPS_vs_nonTFPS_Perc_Rank_Mean_Min_MCC.xlsx in the Supplementary Material), which only includes three non-genomic leading indicator parameter sets. The best performing leading indicator on this basis is the mean cluster logistic growth rate which has a mean normalised MCC of 0·74 (range 0·63 to 0·93). This compares favourably against the highest ranked non-genomic leading indicator parameter set which has a mean normalised MCC of 0·64 (range 0·54 to 0·76).

Sensitivity Analyses

Generation time

The generation time for SARS-CoV-2 may vary across different time periods and for different variants and indeed a range of estimates have been reported during the pandemic. The analysis presented in the main article used a generation time of 6·5 days[5-8]. In order to assess the sensitivity of our methodology to the value chosen for the generation time, we repeated the analysis using a generation time of 5·0 days for the two best leading indicators highlighted in the main article. As shown in Figure S2, there is minimal difference in the leading indicator time series and this made no difference to either the timing of the early warning signals or the number of false positive signals before and after the earliest true positive signal. This sensitivity analysis was also repeated, with the same result, using two different region definitions for geographic aggregation (adm1 and adm2) for the purpose of geo-matching samples in the computation of cluster logistic growth rates, as described in the Methods section.

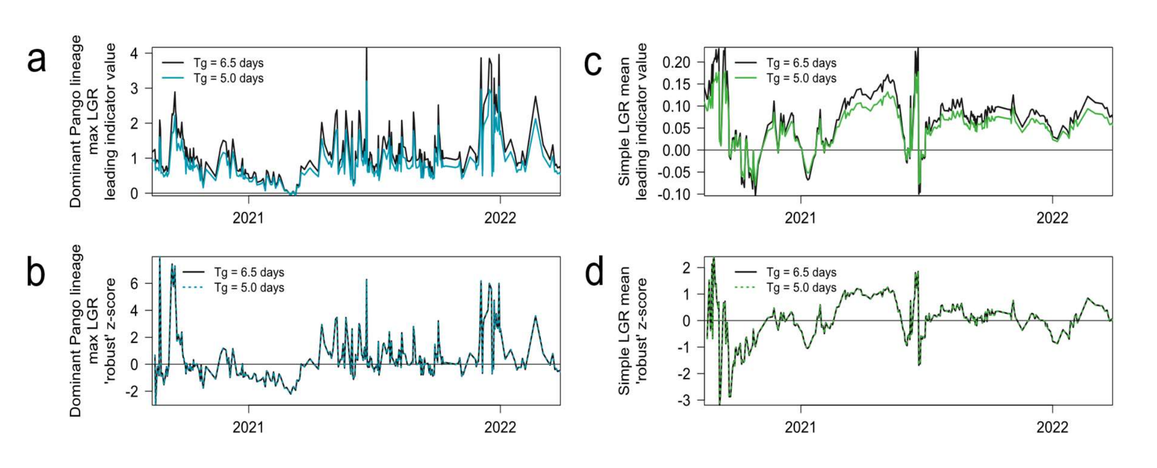

Figure S2: Methodology is not sensitive to value used for generation time. There is a small difference in the time series values for the dominant Pango lineage max logistic growth rate (LGR) leading indicator produced using generation times (T_g) of 6·5 and 5·0 days, as shown in (a), however, when the rolling 'robust' z-score (the time series used to generate early warning signals (EWS)) is computed, there is virtually no difference, as seen in the overlapping plots shown in (b). This situation is repeated for the simple LGR mean leading indicator shown in (c) and (d). The EWS generated using these time series are unchanged by the change of value for generation time input into the Transmission Fitness Polymorphism (TFP) Scanner.

Geographic aggregation

A generalised linear model (GLM) was used to calculate the log odds of a sample being from a cluster of interest compared to a geographically (by country) and temporally matched sample weighted by prevalence, and multiplied by the estimated mean generation time of 6·5 days[5-8] to calculate the relative logistic growth rate per generation for each cluster of interest. To test the sensitivity of the EWS results to the scale of geographic region used in cluster matching we generated EWS using the two best performing leading indicators and parameter sets from the primary analysis but with finer scale geographic matching at administrative level 2 (adm2). The set of genomic sequences was 13·9% smaller given the absence of sufficient metadata to determine the adm2 value. As can be seen from Table S8, the EWS results generated using these two leading indicator and parameter sets are weaker with the finer scale geographic matching, both in terms of lead times and the number of false positives. While it is expected that at smaller scales estimates become more sensitive to stochastic effects and correlated sampling, more extensive analysis would be required to be able to draw a firm conclusion regarding geographic scale given the small number of parameters tested in the sensitivity analysis.

			B.1.177	Alpha	Delta (1)	Delta (2)	Delta (3)	Omicron BA.1	Omicron BA.2
Epidemic wave start (inflection) date			19 Aug 2020	29 Nov 2020	11 May 2021	3 Aug 2021	27 Sep 2021	26 Nov 2021	21 Feb 2022
Dominant Pango lineage max logistic growth rate (LGR) (min cluster age = 7 days, max cluster age = 56 days, min descendants = 20, LGR p-value limit ≤ 0·01, LGR threshold for sub-cluster replacement by parent = 85%, 'robust' z-score threshold for generating EWS = 0·00)									
Earliest True Positive EWS date		Geo. aggregation = country	26 Aug 2020	23 Nov 2020	21 Apr 2021	4 Aug 2021	18 Sep 2021	2 Dec 2021	4 Feb 2022
		Geo. aggregation = administrative level 2	25 Aug 2020	N/A	21 Apr 2021	10 Aug 2021	29 Sep 2021	2 Dec 2021	4 Feb 2022
EWS lead time (days) relative to wave start (inflection) date Lead (-ve) and Lag (+ve)		Geo. aggregation = country	+7	-6	-20	+1	-9	+6	-17
		Geo. agg. = administrative level 2	+6	N/A	-20	+7	+2	+6	-17
Number of False Positives	Prior to earliest True Positive	Geo. aggregation = country	0	0	2	0	0	2	0
		Geo. agg. = administrative level 2	3	N/A	1	0	0	4	0
	After earliest True Positive	Geo. aggregation = country	4	0	0	0	0	0	0
		Geo. agg. = administrative level 2	4	N/A	0	0	0	1	0
Simple LGR mean (min cluster age = 14 days, max cluster age = 56 days, min descendants = 20, no LGR p-value limit, LGR threshold for sub-cluster replacement by parent = 85%, 'robust' z-score threshold for generating EWS = 0·00)									
Earliest True Positive EWS date		Geo. aggregation = country	25 Aug 2020	23 Nov 2020	17 Apr 2021	2 Aug 2021	18 Sep 2021	2 Dec 2021	4 Feb 2022
		Geo. agg. = administrative level 2	25 Aug 2020	18 Dec 2020	17 Apr 2021	2 Aug 2021	26 Sep 2021	2 Dec 2021	4 Mar 2022
EWS lead time (days) relative to wave start (inflection) date Lead (-ve) and Lag (+ve)		Geo. aggregation = country	+6	-6	-24	-1	-9	+6	-17
		Geo. agg. = administrative level 2	+6	+19	-24	-1	-1	+6	+11
Number of False Positives	Prior to earliest True Positive	Geo. aggregation = country	1	0	14	0	0	1	0
		Geo. agg. = administrative level 2	5	0	15	0	0	3	0
	After earliest True Positive	Geo. aggregation = country	4	0	0	0	0	0	0
		Geo. agg. = administrative level 2	0	0	9	0	0	2	0

Table S8: Strongest performing leading indicators using country level geographic aggregation for cluster matching, show weaker performance with finer geographic aggregation. The mean early warning signal (EWS) lead time generated across the seven SARS-CoV-2 epidemic waves in the UK using the dominant Pango lineage max logistic growth rate (LGR) leading indicator fell from 5·4 days to 2·7 days when the geographic aggregation (geo. agg.) for cluster matching was changed to the finer scale administrative level 2. In addition, the total number of false positives increased from 8 (4 before the earliest true positives and 4 after) to 13 (8 before and 5 after). In addition, no EWS was generated for the Alpha wave. The simple LGR mean leading indicator also produced weaker performance at the finer geographic scale: mean lead time of 6·4 days vs. mean lag time of 2·3 days, and 20 false positives (16 before and 4 after) vs 34 (23 and 11).

Supplementary Material References

1 Volz EM, Fitness, growth and transmissibility of SARS-CoV-2 genetic variants. *Nat Rev Genet* 2023. https://doi.org/10.1038/s41576-023-00610-z

2 Volz EM, Boyd O. Transmission Fitness Polymorphism Scanner. Available from: https://github.com/mrc-ide/tfpscanner [Accessed: 30 June 2023]

3 Nicholls SM, Poplawski R, Bull MJ et al. CLIMB-COVID: continuous integration supporting decentralised sequencing for SARS-CoV-2 genomic surveillance. *Genome Biol* 2021; **22**(196). https://doi.org/10.1186/s13059-021-02395-y

4 UK HM Government Department of Health & Social Care. Policy Paper: Coronavirus (COVID-19): Scaling up our testing programmes. Available from: https://www.gov.uk/government/publications/coronavirus-covid-19-scaling-up-testing-programmes/coronavirus-covid-19-scaling-up-our-testing-programmes [Accessed: 30 June 2023]

5 Bi Q, Wu Y, Mei S et al. Epidemiology and transmission of COVID-19 in 391 cases and 1286 of their close contacts in Shenzhen, China: a retrospective cohort study. *Lancet Infect Dis* 2020; **20**(8): 911–919. https://doi.org/10.1016/S1473-3099(20)30287-5

6 Flaxman S, Mishra S, Gandy A et al. Estimating the effects of non-pharmaceutical interventions on COVID-19 in Europe. *Nature* 2020; **584**: 257–261. https://doi.org/10.1038/s41586-020-2405-7

7 Manica M, de Bellis A, Guzzetta G et al. Intrinsic generation time of the SARS-CoV-2 Omicron variant: An observational study of household transmission. *Lancet Reg Health Eur* 2022; **19**: 100446. https://doi.org/10.1016/J.LANEPE.2022.100446

8 Qin W, Sun J, Xu P et al, The descriptive epidemiology of coronavirus disease 2019 during the epidemic period in Lu'an, China: achieving limited community transmission using proactive response strategies. *Epidemiol Infect* 2020; **148**(e132): 1-5. https://doi.org/10.1017/S0950268820001478

9 Fisher RA. The genetical theory of natural selection. Clarendon Press, Oxford, United Kingdom. 1930.

10 Rambaut A, Holmes EC, O'Toole Á et al. A dynamic nomenclature proposal for SARS-CoV-2 lineages to assist genomic epidemiology. *Nat Microbiol* 2020; **5**: 1403–1407. https://doi.org/10.1038/s41564-020-0770-5

11 Wissel C. A universal law of the characteristic return time near thresholds. *Oecologia* 1984; **65**:101-107. https://doi.org/10.1007/bf00384470

12 Scheffer M, Bascompte J, Brock WA et al. Early-warning signals for critical transitions. *Nature* 2009; **461**:53–59. https://doi.org/10.1038/nature08227

13 O'Brien DA, Clements CF. Early warning signal reliability varies with COVID-19 waves. *Biol Lett* 2021; **17**: 20210487. https://doi.org/10.1098/rsbl.2021.0487

14 UK Department of Health & Social Care. Technical Report on the COVID-19 Pandemic in the UK, Chapter 6: testing. 2023. Available from: https://www.gov.uk/government/publications/technical-report-on-the-covid-19-pandemic-in-the-uk/chapter-6-testing [Accessed: 3 July 2023]

15 Hastie T, Tibshirani R. Generalized additive models. *Stat Sci* 1986; **1**(3): 297–310. https://doi.org/10.1214/SS/1177013604

16 UK Health Security Agency. UK Coronavirus Dashboard. Available from: https://coronavirus.data.gov.uk/ [Accessed: 9 May 2022]

17 Wood SN, Pya N, Säfken B. Smoothing Parameter and Model Selection for General Smooth Models. *J Am Stat Assoc* 2016; **111**(516): 1548–1563. https://doi.org/10.1080/01621459.2016.1180986

18 Wood SN. Generalized Additive Models: An Introduction with R (2nd edition). Chapman and Hall/CRC Press, Boca Raton, Florida, USA. 2017.

19 Proverbio D, Kemp F, Magni S, Gonçalves J. Performance of early warning signals for disease re-emergence: A case study on COVID-19 data. *PLoS Comput Biol* 2022; **18**(3): e1009958. https://doi.org/10.1371/journal.pcbi.1009958

20 Hay JA, Kennedy-Shaffer L, Kanjilal S et al. Estimating epidemiologic dynamics from cross-sectional viral load distributions. *Science* 2021; **373**(6552). https://doi.org/10.1126/science.abh0635

21 Phillips M, Quintero D, Butler-Wu S. SARS-CoV-2 Cycle threshold (Ct) values predict future COVID-19 cases. *Open Forum Infect Dis* 2021; **8**(Issue Supplement_1): S31–S32. https://doi.org/10.1093/ofid/ofab466.043

22 Yin N, Dellicour S, Daubie V et al. Leveraging of SARS-CoV-2 PCR Cycle Thresholds Values to Forecast COVID-19 Trends. *Front Med* 2021; **8**: 743988. https://doi.org/10.3389/fmed.2021.743988

23 Lin Y, Yang B, Cobey S et al. Incorporating temporal distribution of population-level viral load enables real-time estimation of COVID-19 transmission. *Nat Commun* 2022; **13**(1155). https://doi.org/10.1038/s41467-022-28812-9

24 Andriamandimby SF, Brook CE, Razanajatovo N et al. Cross-sectional cycle threshold values reflect epidemic dynamics of COVID-19 in Madagascar. *Epidemics* 2022; **38**(100533). https://doi.org/10.1016/j.epidem.2021.100533
25 Google LLC "Google COVID-19 Community Mobility Reports". https://www.google.com/covid19/mobility/ [Accessed: 16 June 2022]
26 Gimma A, Munday JD, Wong KLM et al. Changes in social contacts in England during the COVID-19 pandemic between March 2020 and March 2021 as measured by the CoMix survey: A repeated cross-sectional study. *PLOS Med* 2022; **19**(3): e1003907. https://doi.org/10.1371/JOURNAL.PMED.1003907
27 Dowdy D, D'Souza G. COVID-19 Testing: Understanding the "Percent Positive". 2020. Available from: https://publichealth.jhu.edu/2020/covid-19-testing-understanding-the-percent-positive [Accessed: 3 July 2023]
28 Dahdouh E, Lázaro-Perona F, Romero-Gómez MP, Mingorance J, García-Rodriguez J. Ct values from SARS-CoV-2 diagnostic PCR assays should not be used as direct estimates of viral load. *J Infect* 2021; **82**(3): 426–428. https://doi.org/10.1016/J.JINF.2020.10.017
29 Tom MR, Mina MJ. To Interpret the SARS-CoV-2 Test, Consider the Cycle Threshold Value. *Clin Infect Dis* 2021; **71**(16): 2252–2254. https://doi.org/10.1093/CID/CIAA619
30 Fryer HR, Golubchik T, Hall M et al. Viral burden is associated with age, vaccination, and viral variant in a population-representative study of SARS-CoV-2 that accounts for time-since-infection-related sampling bias. *PLoS Pathog* 2023; 19(8): e1011461. https://doi.org/10.1371/journal.ppat.1011461
31 Matthews BW. Comparison of the predicted and observed secondary structure of T4 phage lysozyme. *Biochim Biophys Acta (BBA) Protein Struct* 1975; **405**(2): 442–451. https://doi.org/10.1016/0005-2795(75)90109-9
32 Chicco D, Jurman G. The Matthews correlation coefficient (MCC) should replace the ROC AUC as the standard metric for assessing binary classification. *BioData Min* 2023; **16**(4): 1–23. https://doi.org/10.1186/s13040-023-00322-4
